# High‐Efficiency Semitransparent Solar Cells Based on Magnetron Sputtered Sb_2_S_3_ Thin Films

**DOI:** 10.1002/advs.202512103

**Published:** 2025-10-17

**Authors:** Pankaj Kumar, Pawan Kumar, Joseph P. Thomas, Alessandro Gradone, Nicola Gilli, Shujie You, Vittorio Morandi, Kam Tong Leung, Alberto Vomiero

**Affiliations:** ^1^ Division of Materials Science Department of Engineering Sciences and Mathematics Luleå University of Technology Luleå SE‐971 87 Sweden; ^2^ WATLab and Department of Chemistry University of Waterloo Waterloo Ontario N2L 3G1 Canada; ^3^ Institute for Nanostructured Materials (ISMN) – CNR section of Bologna Via Piero Gobetti 101 Bologna 40129 Italy; ^4^ Institute for Nanostructured Materials (ISMN) – CNR Strada Provinciale 35 d, n. 9, Montelibretti (RM) Rome 00010 Italy; ^5^ Department of Molecular Sciences and Nanosystems Ca' Foscari University of Venice Via Torino 155 Venezia Mestre 30172 Italy

**Keywords:** antimony sulfide solar cells, average visible transmittance, Sb_2_S_3_, RF magnetron sputtering, semitransparent solar cells, thin film solar cells

## Abstract

Thin‐film solar cells based on wide‐bandgap antimony sulfide (Sb_2_S_3_) offer new possibilities for semitransparent building‐integrated photovoltaics. In this report, impurity‐free Sb_2_S_3_ thin films are prepared by radio frequency magnetron sputtering. The optimized opaque devices obtain a high power conversion efficiency (PCE) of 4.6%. A detailed characterization is conducted to investigate the impact of annealing conditions on the morphology, crystal structure, composition, and optoelectronic properties of Sb_2_S_3_ thin films, which are relevant to photovoltaic performance. Moreover, semitransparent solar cells are fabricated using highly compact Sb_2_S_3_ films with thicknesses of 40, 60, and 80 nm. Using a superstrate device structure (FTO/TiO_2_/Sb_2_S_3_/P3HT‐PEDOT:PSS/Au (≈10 nm)), these solar cells achieve PCEs of 2.0%, 2.6%, and 3.2%, respectively, while maintaining an average visible transmittance (AVT) of 15.5%, 13.5%, and 10.0%. The AVTs of the semitransparent devices are further enhanced by replacing the ultrathin Au top electrode with indium‐doped tin oxide and using wide bandgap inorganic CuSCN as the hole transport layer instead of P3HT‐PEDOT:PSS. Thus, the AVT improves to 20.5% (PCE: 2.0%) for semitransparent solar cells using 60 nm Sb_2_S_3_. This study demonstrates that sputtering is a promising deposition technique for high‐quality ultrathin Sb_2_S_3_ absorbers for semitransparent photovoltaics.

## Graphical abstract

Compact Sb_2_S_3_ films are deposited via RF magnetron sputtering using an Sb_2_S_3_ target. Post‐deposition annealing conditions are optimized to achieve a power conversion efficiency of 4.6%. Semitransparent solar cells are then fabricated with transparent top electrodes, resulting in an average visible transmittance of over 20%.

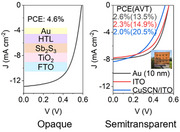

## 1 Introduction

To enhance the effectiveness of photovoltaics as a cleaner renewable energy source, it is crucial to investigate new solar absorber materials and innovative designs. Wide‐bandgap absorber materials in semitransparent solar cell designs can be applied as building‐integrated photovoltaics (BIPV) on unexplored areas of roofs, facades, walls, and windows. Antimony sulfide (Sb_2_S_3_) is a binary chalcogenide absorber that exhibits a high absorption coefficient (10^5^ cm^−1^), a wide optical bandgap of 1.7 eV, and remarkable stability (as it exists naturally as the mineral stibnite). It also has an elemental abundance and relatively low processing temperatures of 300–350 °C (potential for flexible solar cells on polyimide substrates).^[^ ^1^, ^2^ ^]^ A band gap of ≈1.7 eV is also ideal for tandem solar cells in combination with silicon solar cells since the low‐wavelength photons (near IR and IR photons) are transmitted through the Sb_2_S_3_ absorber layer.

For semitransparent Sb_2_S_3_ solar cells, films less than 100 nm thick are required for sufficient transparency. Reducing the thickness of thin‐film solar cells often results in a significant decline in performance. This occurs because a thinner absorber layer increases the surface area‐to‐volume ratio of the Sb_2_S_3_ layer, which amplifies grain boundaries' influence on the solar cells' overall performance. In addition, obtaining a pinhole‐free ultrathin Sb_2_S_3_ film is challenging, especially for solution‐based methods with island‐like nucleation‐growth characteristics and a lack of precise thickness control.^[^ ^3^ ^]^

Magnetron sputtering is a proven technology for the large‐scale production of absorber layers in conventional thin‐film solar cells (copper indium gallium selenide (CIGS) and cadmium telluride (CdTe)). This physical vapor deposition (PVD) method allows for a uniform and compact film with deposition rate control in the range of nanometers. Moreover, a single stable binary phase of Sb_2_S_3_ provides a simple fabrication process for preparing the sputtering target. Sputtered Sb_2_S_3_ films have been realized using two approaches: a) Sputtering of Sb followed by sulfurization and b) Sputtering of Sb_2_S_3_ directly using an Sb_2_S_3_ target followed by post‐annealing.^[^ ^4^, ^5^, ^6^, ^7^, ^8^ ^]^ Both these approaches have obtained power conversion efficiencies (PCEs) of less than 3%, attributed to defects in the Sb_2_S_3_ layer along with the lack of sufficient study on sputtered Sb_2_S_3_ (listed in Table S1, Supporting Information).^[^ ^4^, ^5^, ^6^, ^7^, ^8^, ^9^ ^]^

Post‐deposition annealing is an essential step for obtaining high‐performance Sb_2_S_3_ solar cells. Annealing promotes grain growth and crystallization, reduces bulk defects, and releases the internal stress of the Sb_2_S_3_ film.^[^ ^8^ ^]^ In particular, annealing in the presence of a small amount of sulfur (sulfurization) has been one of the most successful treatments for improving the performance of Sb_2_S_3_ solar cells. This sulfurization step is even more critical for vacuum‐processed Sb_2_S_3_ films to compensate for the possible loss of sulfur during deposition and post‐annealing.^[^ ^8^, ^10^, ^11^, ^12^ ^]^ Deng et al.^[^ ^10^ ^]^ used a sulfur‐atmosphere annealing of Sb_2_S_3_ films prepared by rapid thermal evaporation. The sulfur atmosphere recrystallization step improved the film morphology, decreased the defect concentration (sulfur vacancies and oxides), and resulted in a stoichiometric Sb/S ratio. These improvements led to substantial improvements in PCE, from 4.81% for the control device to 6.25% for the device using the sulfurized Sb_2_S_3_ film. Given the relatively low PCEs reported for sputtered Sb_2_S_3_‐based solar cells (Table S1, Supporting Information), the processing conditions—particularly the post‐annealing treatments—must be carefully optimized. Further investigation is needed to determine how sputtering deposition conditions and post‐deposition annealing treatments affect the performance of sputtered Sb_2_S_3_ thin films, to identify the key parameters limiting photovoltaic performance.

The previous record for PCE (2.89%) using sputtered Sb_2_S_3_ absorber layers was obtained with the well‐established cadmium sulfide (CdS) as an n‐type buffer layer.^[^ ^4^ ^]^ The toxicity of cadmium and the harmful waste produced during the conventional chemical bath deposition (CBD) method highlight the need for greener, nontoxic alternatives to CdS. This transition is crucial for promoting the broader adoption of Sb_2_S_3_ solar cells. Furthermore, unlike a substrate configuration (derived from the conventional CIGS solar cells), which typically employs Mo as the bottom contact, the superstrate configuration uses a transparent bottom contact (such as fluorine‐doped tin oxide (FTO)) as the bottom electrode. The superstate device structure with FTO as the back contact is preferred, as it is compatible with tandem, bifacial, and semitransparent device designs.

In this study, the morphology, chemical composition, and optoelectronic properties of radio frequency (RF) magnetron sputtered Sb_2_S_3_ thin films have been thoroughly investigated. Comprehensive optimization of post‐deposition annealing conditions was performed, resulting in a PCE of 4.6% in a superstrate‐structured Sb_2_S_3_ thin film solar cell. Additionally, semitransparent solar cells with different levels of transparency have been reported, utilizing the precise thickness control in sputtering deposition. This study shows that sputtering is a competitive deposition method for obtaining high‐quality, impurity‐free Sb_2_S_3_ thin films for solar cell applications.

## 2 Results and Discussion

### 2.1 Characterization of Sb_2_S_3_ Films Prepared by RF Magnetron Sputtering on TiO_2_

**Figure 1:**
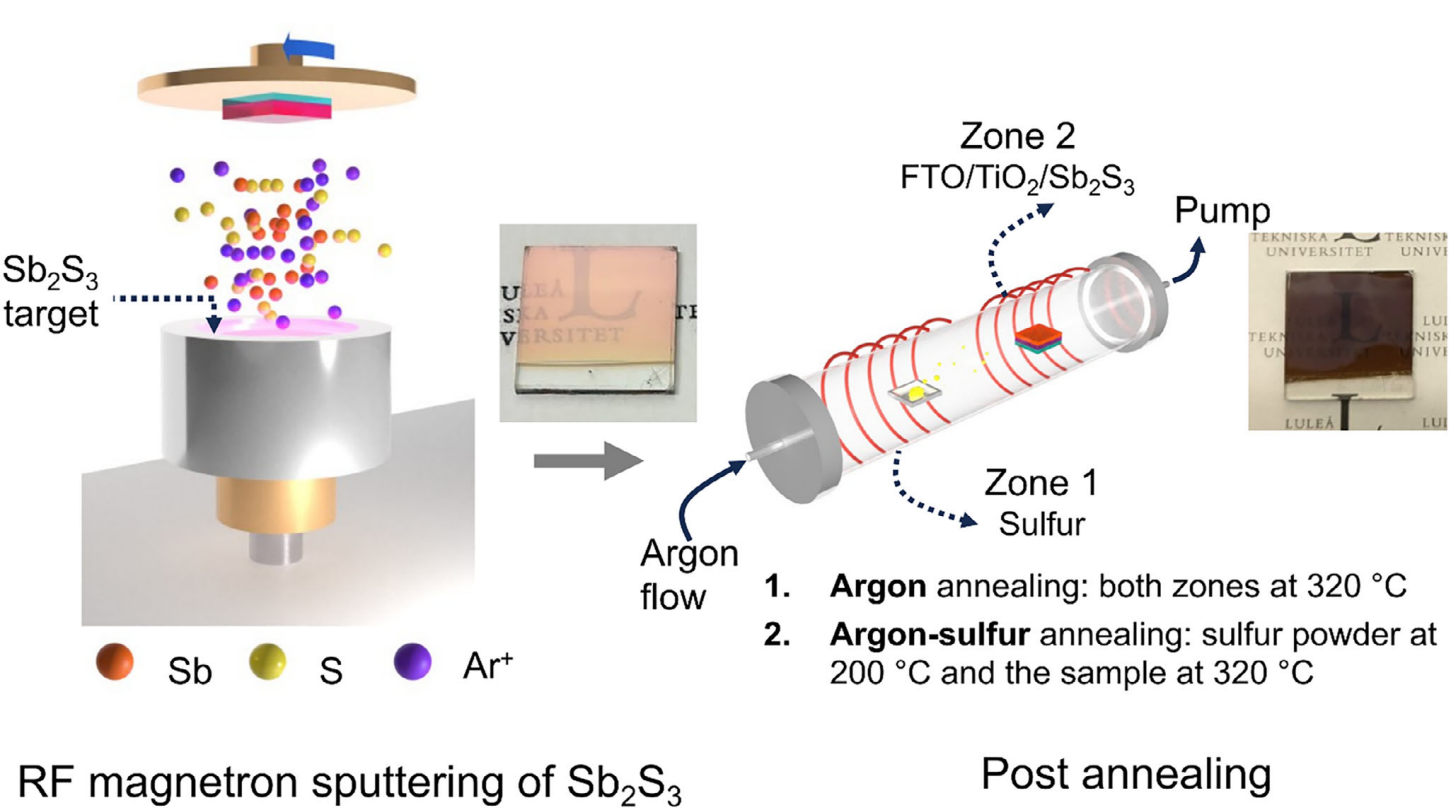
Schematic of Sb_2_S_3_ film preparation. RF sputtering using Sb_2_S_3_ target and post‐annealing treatment with sulfur (“argon‐sulfur”) and without sulfur (“argon”) in an argon‐filled chemical vapor deposition tube. A photograph of a typical argon‐sulfur‐annealed Sb_2_S_3_ film (≈120 nm) on FTO/TiO_2_ is shown on the right, and the as‐deposited film is shown in the middle. Note: the transparent regions on the samples correspond to the areas covered by the sample holder.

**Figure** **1** shows a simplified schematic of RF magnetron sputtering of Sb_2_S_3_ thin film on FTO/TiO_2_ substrates and post‐annealing treatment. The sputtering process was carried out at room temperature (RT) for 15 min at 50 W power and 3 × 10^−3^ mbar working pressure. These conditions were optimized for achieving the best PCEs, which will be discussed later. It is worth noting that deposition at a higher temperature (e.g., 300 °C) gives a crystalline as‐deposited film, as shown in the X‐ray diffraction (XRD) pattern in Figure S1a (Supporting Information). However, the morphology of the films at 300 °C was visibly rough (photographs of depositions at RT, 100, 200, and 300 °C are shown in Figure S1b, Supporting Information) with incomplete coverage of the FTO/TiO_2_ substrates (corresponding scanning electron microscopy (SEM) images are shown in Figure S1c, Supporting Information). The performance of the devices at higher temperatures of 100 and 200 °C was inferior to that deposited at RT (discussed in the photovoltaic performance section). Hereafter, the as‐deposited films will refer to films deposited with substates at RT.

Subsequently, the as‐deposited films were annealed under argon gas protection with or without the addition of sulfur powder. This sulfur‐supplemented annealing significantly improves photovoltaic performance (discussed in the following sections), and similar results have been reported elsewhere.^[^ ^8^, ^10^, ^11^, ^12^ ^]^ As evident from the photographs of the as‐deposited and annealed films (Figure 1), the process yielded a shiny, compact, and microvoid‐free Sb_2_S_3_ film (compactness and absence of microvoids will be evident in SEM and atomic force microscopy (AFM) images) on TiO_2_ (a wide bandgap and green electron transport layer (ETL) alternative to CdS). This is significant because producing compact Sb_2_S_3_ films on bare TiO_2_ using solution‐based methods is challenging.^[^ ^13^ ^]^ Therefore, CdS has been the dominant electron transport/buffer layer for high‐performance Sb_2_S_3_ solar cells for solution‐based methods.^[^ ^14^, ^15^ ^]^ Büttner et al.^[^ ^16^ ^]^ reported dewetting issues even for atomic layer deposition (ALD) of Sb_2_S_3_ on TiO_2_ substrates during annealing at 300 °C for 2 min. As a PVD method involving the ejection of atoms from the target that are deposited onto a substrate, RF magnetron sputtering offers excellent adhesion, uniformity, and density.

**Figure 2:**
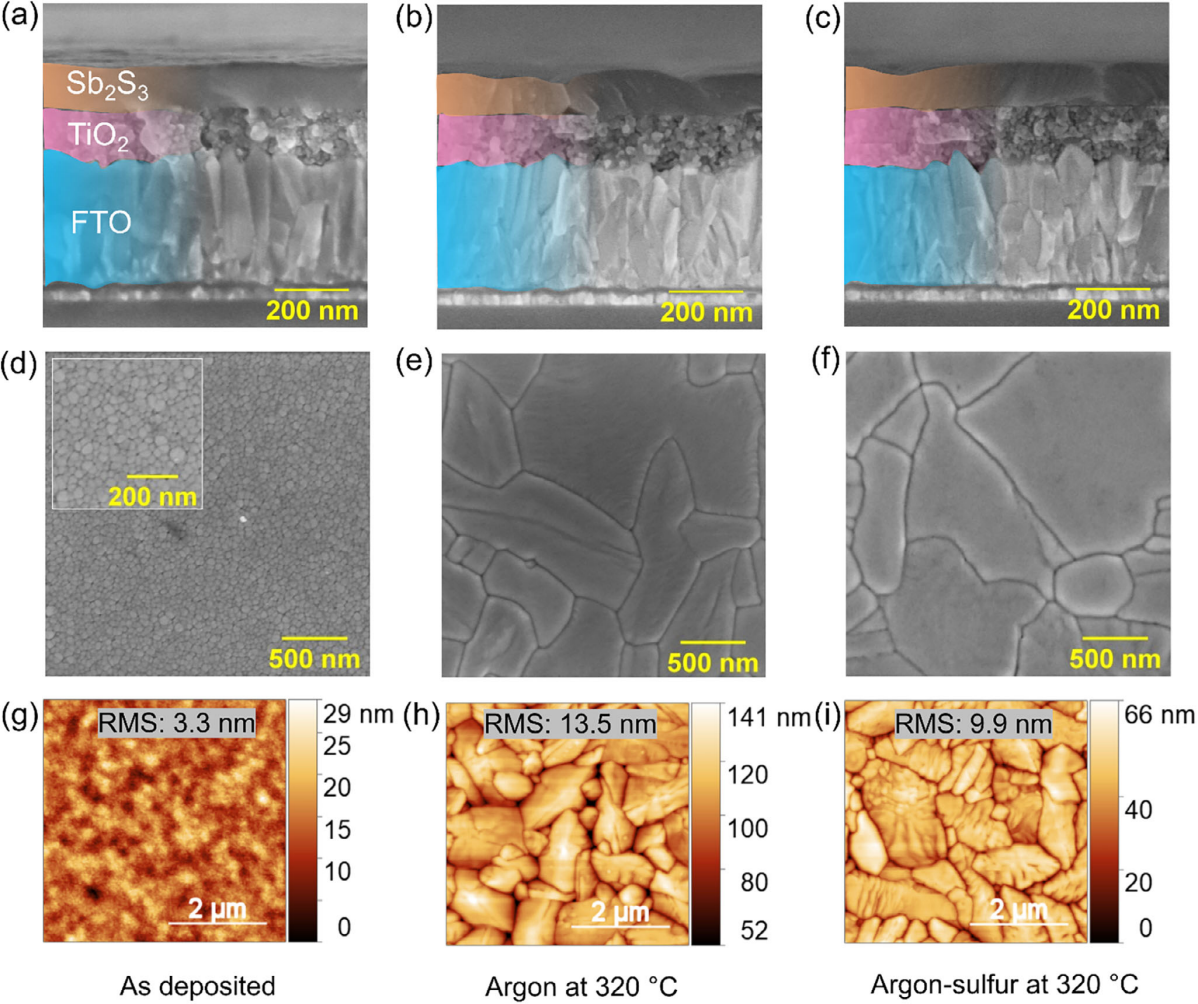
Morphology of sputtered Sb_2_S_3_ thin films. The cross‐section and top‐view SEM images of a,d) as‐deposited, b,e) argon‐annealed, and c,f) argon‐sulfur annealed Sb_2_S_3_ films on FTO/TiO_2_. 2D AFM images of g) as‐deposited, h) argon‐annealed, and i) argon‐sulfur annealed Sb_2_S_3_ thin films deposited on FTO/TiO_2_.

The cross‐section and top‐view morphologies of RF magnetron‐sputtered Sb_2_S_3_ films were analyzed by SEM and AFM. The cross‐section of the deposited film showed intimate contact with the underlying TiO_2_. Annealed films exhibited a slightly rougher morphology due to the crystallization of the Sb_2_S_3_ films, but remained compact and microvoid‐free (**Figure** **2**a–c). In the top‐view SEM image, the as‐deposited films exhibited smooth and compact growth (Figure 2d). The grains measured in the tens of nanometer range (average grain size was calculated as 28 ± 9 nm, as shown in Figure S2a, Supporting Information). Annealed films showed well‐defined grains (sub‐µm in size) (Figure 2e,f). The as‐deposited fine grains seem to coalesce and grow laterally to form compact grains. The grain size distributions for argon and argon‐sulfur annealed films are shown in Figure S2b,c (Supporting Information), respectively. As seen in AFM images (Figure 2h,i), these grains are typically rhombic in geometry.^[^ ^17^, ^18^ ^]^ The as‐deposited film (Figure 2g) with a mirror‐smooth surface showed a root mean square (RMS) roughness of (3.6 ± 0.1) nm. After annealing, both films exhibited increased roughness. However, the film treated with a small amount of sulfur (argon‐sulfur) had a smoother surface (RMS ═ (9.5 ± 0.5) nm) compared to the film annealed in argon (RMS ═ (13.0 ± 0.6) nm). Flat and compact films are beneficial for forming good contact with the hole transport layer (HTL) deposited over the Sb_2_S_3_ film in n‐i‐p solar cells. This would result in a reduction in leakage current and recombination. The annealing environment alters the crystal growth conditions. The addition of a sulfur atmosphere may create a favorable condition (rapid element transfer) for the smaller amorphous grains to recrystallize from the edges laterally, filling the voids more effectively and leaving a smoother morphology.^[^ ^19^ ^]^

**Figure 3:**
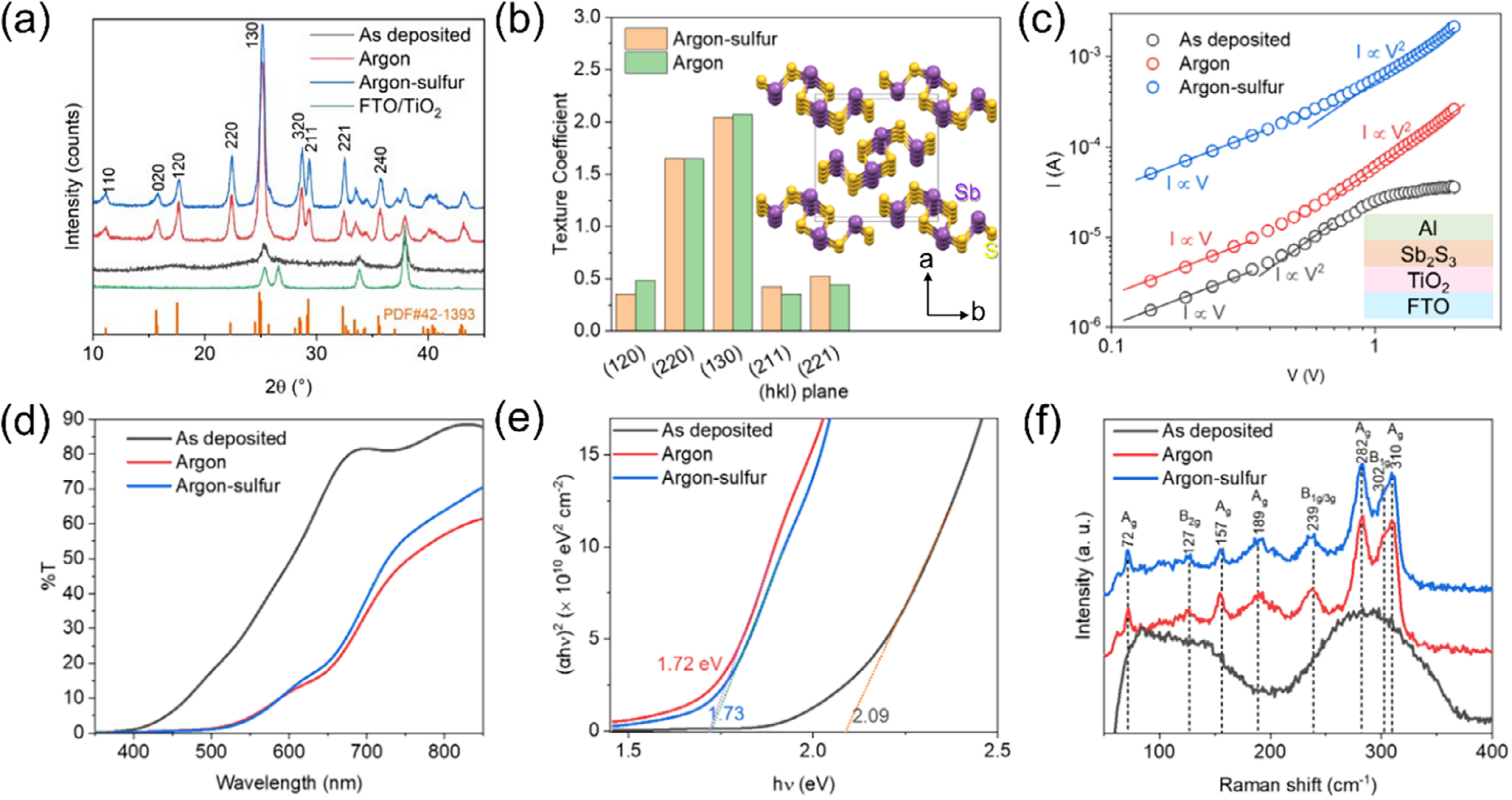
Characterization of Sb_2_S_3_ film prepared by RF sputtering and post‐annealed at 320 °C in argon or argon‐sulfur ambient. a) Grazing incidence X‐ray diffraction patterns of the Sb_2_S_3_ films on FTO/TiO_2_ substrate. b) Texture coefficient of main (hkl) planes. A perspective view of the Sb_2_S_3_ ribbon‐like crystal structure is projected on the c‐axis ([001] direction or a,b plane). c) Double logarithmic current‐voltage (*I–V)* plot for electron‐only devices. Both the ohmic region (slope ═ 1) and the SCLC region (slope ≈ 2) are shown via linear fit lines. (d) Transmittance spectra, (e) Tauc plot, and (f) Raman spectra of the Sb_2_S_3_ films.

Grazing incidence XRD (GIXRD) patterns of the Sb_2_S_3_ films as‐deposited and annealed at 320 °C in argon or argon‐sulfur are shown in **Figure** **3**a. For the as‐deposited films, apart from a broad peak ≈15°–17°, no prominent sharp peaks belonging to Sb_2_S_3_ (except the FTO/TiO_2_ substrate peaks) can be seen, suggesting an amorphous nature. This is consistent with most of the previous reports for as‐deposited (at RT) and unannealed films.^[^ ^9^, ^20^ ^]^ Sharp peaks appear in the argon and argon‐sulfur annealed films, corresponding to the stibnite Sb_2_S_3_ (JCPDS #42‐1393), suggesting pure‐phase formation without evident impurities. The dominant peaks (120), (220), (130), (211), and (221) were analyzed to calculate the texture coefficient (TC), shown in Figure 3b. The TC calculation procedure is reported elsewhere.^[^ ^20^ ^]^ Sb_2_S_3_ exhibits an orthorhombic phase characterized by an infinite arrangement of 1D ribbons along the [001] (or c) direction (a c‐axis projection of crystal structure is shown in Figure 3b). Therefore, charge transport along the c‐axis is unhindered. The (hk1) planes, thus, are preferred for efficient charge transport and reduced recombination losses. Annealing in the presence of sulfur leads to a slight improvement in the TC of the quasi‐vertical (211) and (221) planes and a decrease in the TC of near‐parallel (120) and (130) planes. Qiu et al.^[^ ^12^ ^]^ also observed that a sulfur‐supplemented vapor transport deposition promotes a preferential (hk1) orientation of the Sb_2_S_3_ film. This observation is consistent with other reports and is related to a change in the crystal growth process.^[^ ^12^, ^19^ ^]^ Nevertheless, (hk0) planes still dominate in both films. It should be noted that some reports using the sputtering method had indeed obtained in‐situ (hk1) planes by controlling the growth/annealing temperature.^[^ ^4^, ^6^, ^21^ ^]^ In the Sb_2_S_3_ films deposited at a substrate temperature of 300 °C in this study (Figure S1a, Supporting Information), a preferred (hk1) orientation was not seen, but the morphology degraded significantly (as discussed earlier). Apparently, the crystallization orientation also depends on the substrate nanostructure and the Sb_2_S_3_ film thickness.^[^ ^22^ ^]^ Therefore, it requires optimization of growth/annealing conditions to obtain both compact and (hk1)‐oriented films, simultaneously.

Electron‐only devices (FTO/TiO_2_/Sb_2_S_3_/Al) were fabricated to evaluate the comparative charge transport behavior (electron mobilities) of the Sb_2_S_3_ films as a function of the annealing conditions. The ohmic and space charge regions can be identified in the double logarithmic current‐voltage plot in Figure 3c. The electron mobilities (µ_e_) were calculated using the Mott–Gurney equation,^[^ ^23^ ^]^ which describes the space charge limited current (SCLC) in the current density‐voltage (*J–V)* curve of a single carrier device in the dark:

(1) $$J=\frac{9}{8}\varepsilon_{r}\varepsilon_{0}\mu_{e}\frac{V^{2}}{L^{3}}$$

where ε_0_ is the permittivity of free space, ε_*r*_═ 7.1,^[^ ^24^ ^]^ is the dielectric constant of Sb_2_S_3_, V is the voltage, and L is the thickness of the film (≈250 nm, measured by cross‐section SEM). As shown in Figure S3 (Supporting Information) (J^−2^ vs V plot as per Equation 1), the electron mobility of Sb_2_S_3_ film increased by an order of magnitude upon annealing. Sb_2_S_3_ film without annealing showed poor electron mobility of 5.5 × 10^−6^ cm^2^ V^−1^ s^−1^. The mobility improved to 2.2 × 10^−5^ and 1.3 × 10^−4^ cm^2^ V^−1^ s^−1^ for argon and argon‐sulfur annealed devices, respectively. The increase in mobility from amorphous to crystalline films is attributed to reduced carrier scattering due to the larger grain sizes in the annealed films.^[^ ^25^ ^]^ The higher currents in the ohmic region (slope ═ 1 region in Figure 3c) also suggest higher carrier concentration or lower deep‐defect concentrations (which capture charge carriers) for argon‐sulfur annealed Sb_2_S_3_ thin films.^[^ ^11^ ^]^

The transmission spectra of as‐deposited and annealed films are shown in Figure 3d. The bandgaps were calculated using the Tauc plot, assuming a direct bandgap (described in the experimental section) (Figure 3e). As deposited, amorphous Sb_2_S_3_ films showed a bandgap (E_g_) of 2.1 eV, which reduces to 1.7 eV for the annealed films. These values agree with the previous reports for amorphous and crystalline Sb_2_S_3_ films.^[^ ^26^, ^27^ ^]^ Also, in Figure 3e, the amorphous film shows an exponential tailing (absorption below the band edge), suggesting high defect density due to disorder or dangling bonds at the surfaces of the nanocrystallites.^[^ ^24^, ^25^ ^]^ The dangling bonds and defects are also expected to be present to some extent at the grain boundaries, even for the annealed films. In crystalline films, defects in the crystalline structure, such as atom vacancies or displacements, create trap states within the bandgap.^[^ ^25^ ^]^ A bandgap of 1.7 eV is ideal for tandem application in combination with silicon solar cells. The decreased optical transmission of Sb_2_S_3_ thin films after annealing can be due to improved crystallinity and increased grain size (as seen in the XRD and SEM). The decrease in the band gap is likely due to an amorphous‐to‐crystalline transition, as demonstrated by XRD of Sb_2_S_3_ after annealing.^[^ ^28^, ^29^ ^]^

Raman spectra (shown in Figure 3f) confirm the amorphous nature of the as‐deposited Sb_2_S_3_ films, the crystallization induced by annealing, and the phase purity of the annealed Sb_2_S_3_ films. The amorphous as‐deposited films showed a broad peak centered ≈285 cm^−1^, which, upon annealing, splits into three peaks at 282, 303, and 310 cm^−1^. Moreover, the additional peaks at 72, 127, 157, 189, and 239 cm^−1^ can all be assigned to Sb_2_S_3_. The absence of Raman modes at ≈253 cm^−1^ indicates the absence of detectable oxide phases.^[^ ^22^, ^28^, ^29^, ^30^ ^]^

**Figure 4:**
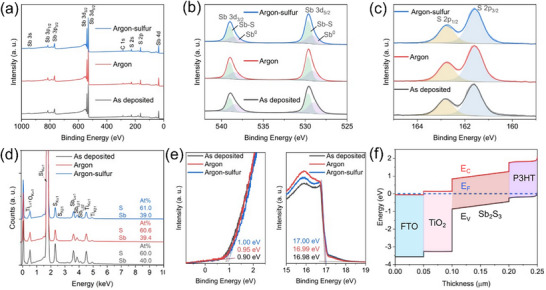
XPS spectra of the as‐deposited, argon‐annealed, and argon‐sulfur‐annealed films showing a) Survey spectra, b) High‐resolution Sb 3d region, and c) S 2p region. d) EDS spectra of the Sb_2_S_3_ films on Si/TiO_2_ substrates. e) UPS spectra of Sb_2_S_3_ thin films using a photon source with energy of 21.22 eV. Left: the UPS spectra in the valence band region. Right: the secondary electron cut‐off region. f) SCAPS‐1D calculated energy band diagram of the films used in the solar cell device at equilibrium (for argon‐sulfur annealed Sb_2_S_3_ films).

Impurities in the Sb_2_S_3_ films influence the defect properties and solar cell performance. X‐ray photoelectron spectroscopy (XPS) characterization was employed to investigate the chemical compositions of these films. Survey spectra are shown in **Figure** **4**a, where major peaks can be assigned to Sb and S atoms. XPS peaks at binding energies (BEs) of 538.9 and 529.6 eV correspond to Sb 3d_3/2_ and Sb 3d_5/2_ of Sb^3+^ in Sb_2_S_3_, respectively, regardless of annealing conditions (Figure 4b). Minor peaks at lower binding energies (538.1 and 528.7 eV in sulfur‐annealed films, for example) correspond to the elemental state (Sb^0^). They are present even in the as‐deposited films and are attributed to the Ar^+^ sputter‐reduced metallic Sb atoms during deposition or sputter etching prior to XPS measurement.^[^ ^5^, ^30^, ^31^ ^]^ The metallic Sb‐Sb bonds appear to be present only on the surface since no prominent peaks related to Sb metal were found in XRD. They are detected in XPS (penetration depth 2–3 nm) but not in XRD patterns because no large crystallites are formed. Also, no prominent oxide peaks were detected, suggesting either the absence or a minimal amount of oxide impurity in the films. This is further supported by the absence of O 1s KLL peak at 984 eV (Figure 4a).^[^ ^16^ ^]^ Also, the peaks at a binding energy of 162.7 and 161.5 eV (for argon‐sulfur annealed film, shown in Figure 4c), respectively, are ascribed to S 2p_1/2_ and S 2p_3/2_. Thus, from both Raman and XPS analyses, regardless of annealing conditions, the Sb_2_S_3_ films produced via RF magnetron sputtering are free from oxides or impurities—an advantage over solution‐based methods.^[^ ^15^ ^]^

Qualitative analysis of elemental composition was done by energy‐dispersive X‐ray spectroscopy (EDS). The corresponding representative EDS spectra are shown in Figure 4d. The as‐deposited films showed stoichiometric elemental composition (S/Sb = 1.50). This is an advantage of using a binary Sb_2_S_3_ target instead of an Sb target (which needs careful sulfurization to convert all Sb atoms). The films annealed in argon and argon‐sulfur environments also showed the near stoichiometric composition of 1.54 and 1.56, respectively. A small S‐excess has been reported to decrease the resistivity of Sb_2_S_3_ by more than one order.^[^ ^10^ ^]^ The XPS quantitative analysis showed a similar trend with S/Sb ratios calculated to be 1.29, 1.38, and 1.40, respectively, for the as‐deposited, argon‐annealed, and argon‐sulfur‐annealed Sb_2_S_3_ films (Figure S4, Supporting Information). The presence of metallic Sb near the surface might be the reason for the reduced relative S concentration in XPS analysis compared to that of the bulk composition measured using EDS.

The solar cell device performance is influenced by the relative energy level alignment of the ETL and HTL with respect to those of the Sb_2_S_3_ film. The energy level positions were determined using ultraviolet photoelectron spectroscopy (UPS) (Figure 4e). The binding energy cutoff edge (E_cutoff_) values near the surfaces of Sb_2_S_3_ films annealed in argon and argon‐sulfur were similar (16.99 eV and 17.00 eV, respectively). By extrapolating the linear region of low binding energy, the energy gaps (E_onset_) between the valence band maximum and Fermi energy were 0.95 and 1.00 eV, respectively. Consequently, the conduction band (E_C_), valence band (E_V_), and Fermi levels (E_F_) were determined from the E_cutoff_ and E_onset_ values using the following equations:

(2) $$E_{F}=h\nu -E_{\mathrm{cuto}\mathrm{ff}}$$

(3) $$E_{v}=E_{F}+E_{\mathrm{onset}}$$

(4) $$E_{c}=E_{v}\;-E_{g}$$

where *h*ν is the energy of the UV photoelectron (21.22 eV), and E_g_ is the bandgap of the films. Figure 4f shows the relative energy level alignment of thin films (w.r.t. argon‐sulfur annealed Sb_2_S_3_ film) in the solar cell device structure. The calculated E_C_ and E_V_ values are listed in Table S2 (Supporting Information). The Fermi levels of both the annealed films are situated near the midgap, indicating a nearly intrinsic nature. TiO_2_ acts as an electron transport/hole‐blocking layer with its deep E_V_ of −7.62 eV and E_C_ of −4.20 eV (UPS spectra and E_g_ values are shown in Figure S5a–c, Supporting Information). P3HT‐PEDOT:PSS bilayer served as hole transport/collection layer with the work function (≈−5.0 eV) close to the E_V_ of Sb_2_S_3_ and E_F_ of top gold contact (−5.1 eV).^[^ ^16^ ^]^

### 2.2 Photovoltaic Performance

**Figure 5:**
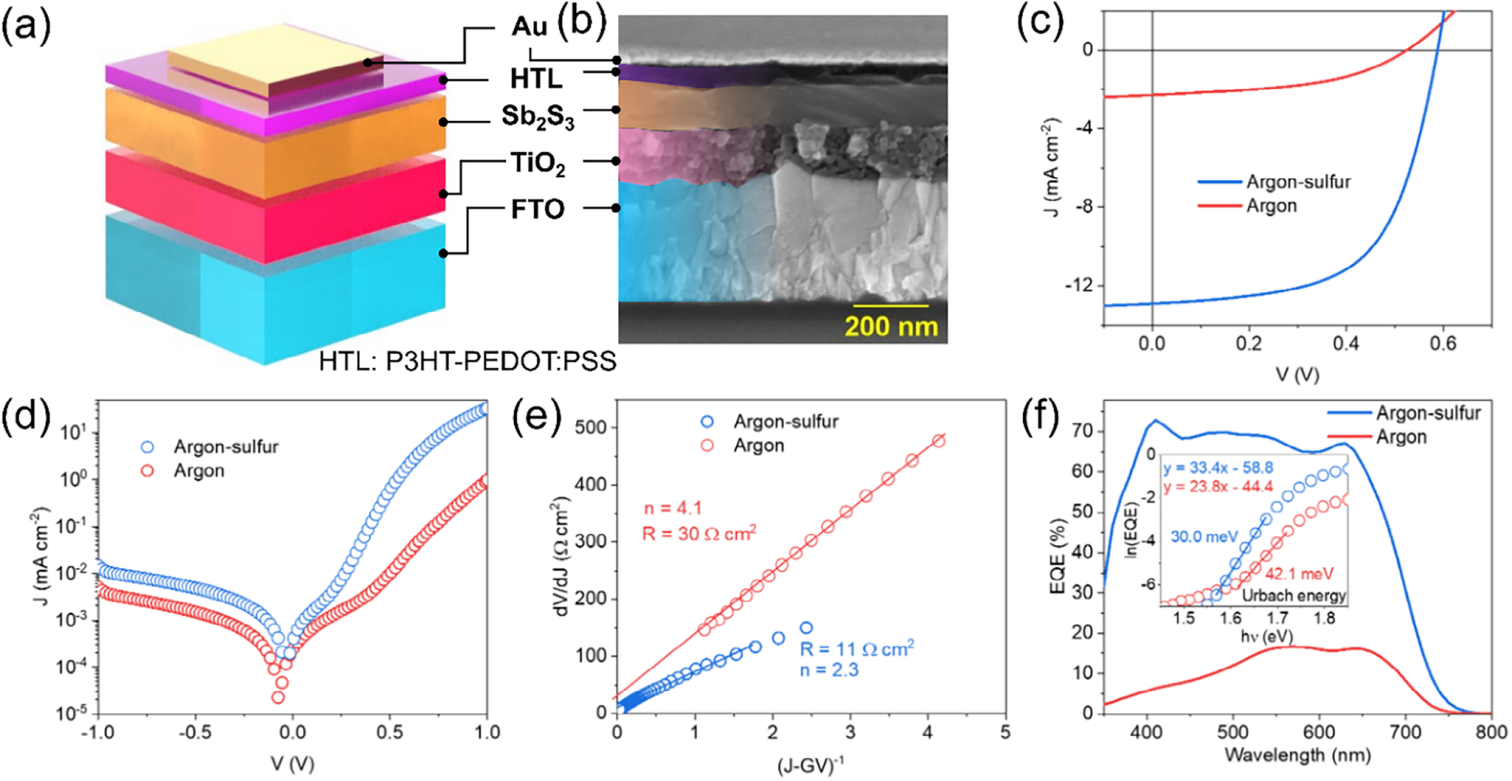
Solar cell performance characterization. a) Device structure and b) cross‐section image of a representative solar cell device using argon‐sulfur annealed Sb_2_S_3_ absorber. c) *J–V* curves of solar cells based on Sb_2_S_3_ films annealed in argon or argon‐sulfur atmosphere. d) Semilog dark *J–V* plot, and e) dV/dJ vs 1/J plot for series resistance and ideality factor calculation from the dark curves. f) EQE spectra with linear fit for Urbach energy (E_U_) calculation in the inset.

The influence of annealing conditions on the photovoltaic performance was tested for the solar cells fabricated in a typical planar superstrate device configuration (n‐i‐p) of FTO/TiO_2_/Sb_2_S_3_/P3HT‐PEDOT:PSS/Au (**Figure** **5**a) with TiO_2_ and P3HT‐PEDOT:PSS as the electron and hole transport layers, respectively. The cross‐sectional SEM image of a typical solar cell (shown in Figure 5b) indicates that the Sb_2_S_3_ absorber layer is uniform and compact, with a thickness of (120 ± 10) nm. The thickness of the absorber layers can be finely tuned simply by varying the sputtering duration, as shown in the progressive thickness growth in SEM cross‐section images (Figure S6a–e, Supporting Information) and the linear relation of thickness vs sputtering time in Figure S3f (Supporting Information). A series of device optimizations were performed for argon‐sulfur annealed Sb_2_S_3_ solar cells to obtain the maximum PCE (as‐deposited and argon‐annealed devices showed poor performance, as discussed later). These include: a) thickness of the Sb_2_S_3_ absorber layer (Figure S7 and Table S3, Supporting Information), b) annealing temperature (zone 2, FTO/TiO_2_/Sb_2_S_3_ substrate) (Figure S8 and Table S4, Supporting Information), c) annealing duration (Figure S9 and Table S5, Supporting Information), d) sulfur amount (Figure S10 and Table S6, Supporting Information), e) sulfur temperature (Zone 1) (Figure S11 and Table S7, Supporting Information), f) substrate temperature during sputtering (Figure S12 and Table S8, Supporting Information), and g) sputtering working pressure (Figure S13 and Table S9, Supporting Information).

The *J–V* curve of the champion solar cells using annealed Sb_2_S_3_ absorber layers is shown in Figure 5c, and the corresponding performance parameters are illustrated in **Table** **1**. Solar cells using Sb_2_S_3_ annealed without sulfur (other conditions were the same as the champion argon‐sulfur devices) suffered significant losses in short‐circuit current density (J_SC_) and fill factor (FF), resulting in a low PCE of 0.6% (average: 0.5% ± 0.01%). Solar cells with Sb_2_S_3_ film annealed in the presence of a small amount of sulfur showed significantly improved device performance, achieving a PCE of 4.6% (average: 4.4% ± 0.1%). Similar PCEs were obtained in both forward and reverse scans (Figure S14, Supporting Information), revealing good bias stability without hysteresis. On the other hand, the as‐deposited Sb_2_S_3_ film showed an extremely low PCE of 0.02% with extremely low short circuit currents (the *J–V* curve and performance parameters are shown in Figure S15 and Table S10, Supporting Information, respectively). The statistical plots of the devices show a direct comparison of the photovoltaic parameters vs annealing conditions in Figure S16 and Table S11 (Supporting Information). The stability of the champion argon‐sulfur devices was tested in dark ambient conditions without encapsulation. The devices retained >95% PCE after 30 days (PCE variation is shown in Figure S17, Supporting Information), suggesting potential for real‐life applications. A good coverage of HTL on a smooth Sb_2_S_3_ film probably helps block water and oxygen molecules.^[^ ^32^ ^]^

**Table 1:** Device performance parameters of solar cell devices based on Sb_2_S_3_ films annealed in argon vs argon‐sulfur ambient.

	*J*_SC_ [mA cm^−2^]	*V*_OC_ [mV]	FF [%]	PCE [%]
Argon	2.2 ± 0.1 (2.3)	524 ± 9 (524)	46.6 ± 0.5 (46.8)	0.5 ± 0.01 (0.6)
Argon‐sulfur	12.9 ± 0.3 (12.9)	582 ± 14 (587)	58.9 ± 0.5 (60.2)	4.4 ± 0.1 (4.6)

The average and standard deviation of the top 8 cells for each condition are reported. In parentheses, the parameters of the champion devices are reported. The thickness of the absorber layer was ≈120 nm.

The dark *J–V* curves of the solar cells were analyzed to determine the reason for the drastic improvement in photovoltaic performance with the addition of a small amount of sulfur during annealing. Compared to argon‐annealed devices, the argon‐sulfur showed higher dark currents in the forward‐bias region (Figure 5d), suggesting better charge transport across the n‐i‐p junction. Further analysis of the *J–V* curves was carried out to study the effects of annealing conditions on the properties and photovoltaic performance of the solar cells. The solar cell *J–V* curves were modeled by a single exponential diode equation^[^ ^33^ ^]^:

(5) $$J=J_{0}exp\left[\frac{q}{nkT}\left(V-RJ\right)\right]+GV-J_{L}$$

where J_0_ represents the diode reverse saturation current, q is the elementary electron charge, n is the diode ideality factor, and k and T are the Boltzmann constant and device temperature, respectively. R and G are series resistance and shunt conductance, respectively. J_L_ = 0 for dark current analysis. The plots of dV/dJ (Figure 5e) (J−GV)^−1^ are used to calculate n and R. The shunt conductance G is calculated from dJ/dV vs V curves in Figure S18a (Supporting Information). The ideality factor of the argon‐sulfur device is reduced from 4.1 to 2.3, indicating a reduction in the defect‐assisted recombination. The series resistance (30 Ω‐cm^−2^) was calculated to be almost three times higher for argon‐only annealed devices (vs 11 Ω‐cm^−2^ for argon‐sulfur annealed devices). The increased R might be the result of lower effective mobility of carriers in the Sb_2_S_3_ film across the device thickness (also supported by SCLC mobility values shown in Figure 3c and Figure S3, Supporting Information). High series resistance results in a lowering of the FF and a loss of current (J_SC_) in the corresponding device. The diode saturation current density J_0_ (determined from ln(J) vs V−RJ plot in Figure S18b, Supporting Information) decreased slightly for argon‐sulfur annealed devices. Lower open circuit voltage (V_OC_) in argon‐annealed devices can be attributed to both higher ideality factors and higher diode recombination currents according to the relation^[^ ^19^ ^]^:

(6) $$V_{OC}=\frac{nkT}{q}ln\left(\frac{J_{SC}}{J_{o}}+1\right)$$

V_OC_ loss originates from non‐radiative recombination, either from the interface or defects in the bulk Sb_2_S_3_.^[^ ^34^ ^]^ Since the band alignment and the interfaces for both devices are similar, the V_OC_ loss is probably related to the deep defects in the absorber layer.

In addition, the defect states of Sb_2_S_3_ were calculated from the dark *J–V* curves. Figure S19 (Supporting Information) shows the log‐log plot of dark *J–V* curves for the argon and argon‐sulfur devices. The logarithmic *J–V* curve generally exhibits three regions, each indicating different mechanisms: a) at low voltage, ohmic behavior; b) at intermediate voltage, the trap‐filled limited (TFL) region; and c) at high voltage, a trap‐free, child region. As the voltage approaches the kink point in the TFL region, the current suddenly rises, indicating that the injected carriers fully occupy the trap states. The V_TFL_ voltages measured were 0.42 V for the argon annealed device and 0.23 V for the argon‐sulfur annealed device. The trap state density (N_trap_) was determined using the following equation^[^ ^35^ ^]^:

(7) $$N_{trap}=\frac{2\varepsilon_{r}\;\varepsilon_{0}V_{TFL}}{qL^{2}}\;$$

where ε_0_ is the permittivity of free space, ε_*r*_═ 7.1,^[^ ^24^ ^]^ is the dielectric constant of Sb_2_S_3,_ q is the elementary electron charge, and L is the thickness of the Sb_2_S_3_ film (120 nm). The obtained N_trap_ values for argon and argon‐sulfur annealed devices were 2.1 × 10^16^ and 1.2 × 10^16^ cm^−3^_,_ respectively. The lower N_trap_ value for devices annealed in a sulfur ambient is attributed to the better quality of the Sb_2_S_3_ film, with fewer traps and defects.^[^ ^4^ ^]^

The loss in J_SC_ is evident from the external quantum efficiency (EQE) curve shown in Figure 5f, although the absorption edges are similar. The loss in EQE over the entire wavelength suggests that the device performance is limited by charge transport resistance throughout the bulk (or low carrier mobility, as discussed above).^[^ ^36^ ^]^ The Urbach energy (E_U_) measures local defects associated with material structural imperfections. These defects and lack of crystalline long‐range order create a tailing of the density of states, which is also responsible for recombination and decreased V_OCs_.^[^ ^37^, ^38^, ^39^ ^]^ This is seen in the long wavelength >750 nm, where there is an exponential decay in EQE due to the presence of band tails (quantified by E_U_). The exponential absorption beyond the absorption edge region is described by $\alpha \left(E\right)=\alpha_{0}\exp\left(E/E_{U}\right)$.^[^ ^39^, ^40^ ^]^ The experimental E_U_ can be calculated from the inverse slope of the linear portion of the ln(EQE) vs hν plot near the E_g_.^[^ ^41^ ^]^ The graph of (hν×ln(1‐EQE))^2^ vs hν was used to calculate the E_g,EQE_, as shown in Figure S20 (Supporting Information). The E_g,EQE_ values are similar to those obtained from transmittance measurements. The inset of Figure 5f presents E_U_ values for the devices using the Sb_2_S_3_ films annealed in argon or argon‐sulfur ambient. A reduction in E_U_ (30.0 vs 42.1 meV) suggests a decrease in non‐radiative recombination losses due to bulk disorder defects in argon‐sulfur annealed Sb_2_S_3_ films, thus improving the V_OC_ in the corresponding device. Energetic sulfur vapor may have promoted structural ordering or reduction in deep defects during crystallization.^[^ ^19^ ^]^ For reference, high‐performance CdTe and CIGS films show E_U_ values in the 10–30 meV range.^[^ ^42^ ^]^

**Figure 6:**
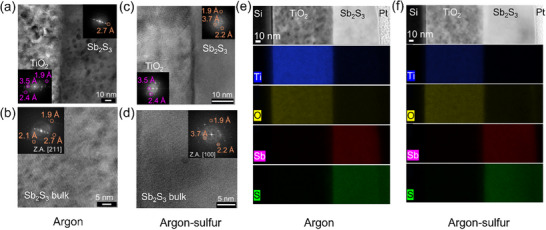
HRTEM images showing TiO_2_/Sb_2_S_3_ interface and Sb_2_S_3_ bulk regions for a,b) argon annealed and c,d) argon‐sulfur annealed Sb_2_S_3_ films. HAADF images and corresponding elemental STEM‐EDS maps for e) argon‐annealed and f) argon‐sulfur annealed films. The top layers are protective Pt coatings added during sample preparation. Silicon was used as a substrate for cross‐section sample preparation. Z. A. in (b,d) refers to zone axis alignment to [211] and [100], respectively.

High‐resolution transmission electron microscopy (HRTEM) was employed to study the potential nanocrystalline structural variation of the Sb_2_S_3_ films due to sulfurization. HRTEM images of the TiO_2_/Sb_2_S_3_ interface and bulk regions for argon‐annealed Sb_2_S_3_ films are shown in **Figure** **6**a,b, respectively. The corresponding images for the argon‐sulfur annealed films are shown in Figure 6c,d, respectively. The crystal structures, investigated by fast Fourier transforms (FFTs), correspond to Sb_2_S_3_ and TiO_2_ (anatase) crystal structures. Specifically, the d‐spacings of 0.35, 0.24, and 0.19 nm belong to the (101), (103), and (200) planes of anatase TiO_2_. On the other hand, for Sb_2_S_3_, d‐spacings of 0.37, 0.27, 0.21, 0.22, and 0.19 nm correspond to (013), (03‐3), (1‐42), (025), and (053) planes, respectively. This indicates that pure‐phase and crystalline Sb_2_S_3_ films were obtained regardless of the presence of sulfur during annealing. Thus, the HRTEM analysis complements the results obtained from XRD, XPS, and Raman measurements.

High‐angle annular dark‐field scanning transmission electron microscopy (HAADF‐STEM) images and energy‐dispersive X‐ray spectroscopy (EDX) mapping were conducted to elucidate the vertical elemental distribution in argon and argon‐sulfur‐annealed Sb_2_S_3_ films. Figure 6e,f show the STEM‐HAADF cross‐sectional images along with EDS elemental mapping for the argon and argon‐sulfur annealed films, respectively. Both TiO_2_ and Sb_2_S_3_ films showed a sharp contrast. The STEM–EDS analysis shows that Sb and S are evenly distributed throughout the film, even in the film annealed with sulfur. Some diffusion of sulfur into the protective Pt layer is a result of the Pt coating method (decomposing a precursor using the e‐beam of the SEM column), which can cause some sulfur to diffuse toward the platinum. However, this does not affect the chemistry of the internal layers, as it remains confined to the surface only. Specifically, the sulfur annealing did not result in an excess of sulfur on the surface. Therefore, annealing in a sulfur‐rich environment mainly influenced the defect chemistry.

The photovoltaic performance enhancement (especially V_OC_) due to sulfur‐supplemented annealing (S‐rich condition) seems to be due to the suppression of deep defects (V_S_, Sb_i_)^[^ ^11^ ^]^ in the bulk of the Sb_2_S_3_ absorber.^[^ ^10^ ^]^ The passivation of these deep defects enhances charge extraction and reduces carrier recombination. Multiple groups have consistently reported this observation, using deep‐level transient spectroscopy and thermal admittance spectroscopy to quantify the reduction in deep defect density and the improvement in carrier lifetimes under S‐rich conditions.^[^ ^10^, ^21^, ^42^, ^43^, ^44^, ^45^ ^]^

**Table** **2** compares the top PCEs from different solutions and vacuum‐based deposition methods for Sb_2_S_3_ solar cells, including sputtering. The solution‐based Sb_2_S_3_ solar cells still outperform those based on vacuum. Solution‐based methods usually deliver compact Sb_2_S_3_ films with large grains.^[^ ^45^ ^]^ However, deposition of ultrathin Sb_2_S_3_ films using solution processing, especially on bare TiO_2_ has been challenging, and film thickness is usually uncontrollable.^[^ ^13^, ^46^ ^]^ Among the vacuum‐based deposition methods, closed space sublimation (CSS), rapid thermal evaporation (RTE), and vapor transport deposition (VTD) have shown promising results with PCEs approaching 6%. However, the high deposition temperatures make them inherently vulnerable to surface oxidation and sulfur loss (due to the high saturated vapor pressure of sulfur).^[^ ^10^ ^]^ The thickness control is not as good as that achieved with sputtering using the Sb_2_S_3_ target. ALD, which allows for the greatest precision in controlling thickness, is relatively slow and requires the use of toxic H_2_S during the deposition process.^[^ ^47^ ^]^

**Table 2:** A comparison of photovoltaic parameters of Sb_2_S_3_ solar cells prepared via different methods.

	Method	Device configuration	*J*_SC_ [mA cm^−2^]	*V*_OC_ [mV]	FF [%]	PCE [%]	Refs.
Solution	FCA^a)^	FTO/TiO_2_/Sb_2_S_3_/Spiro/Au	17.2	720	57.2	7.1	[49]
	CBD	FTO/CdS/Sb_2_S_3_/Spiro/Au	18.3	763	57.7	8.1	[50]
	HT^b)^	FTO/SnO_2_‐NH_4_F/CdS/Sb_2_S_3_/PbS‐EDT/Au	17.9	712	64.8	8.3	[48]
Vacuum	CSS^c)^	FTO/CdS/Sb_2_S_3_/Spiro‐OMeTAD/Au	15.6	739	51.8	5.81	[51]
	RTE^d)^	FTO/TiO_2_/Sb_2_S_3_/Au	16.9	683	54.1	6.25	[10]
	VTD^e)^	ITO/CdS/Sb_2_S_3_/Au	15.7	710	43.2	4.7	[52]
	ALD^f)^	FTO/TiO_2_‐c/Sb_2_S_3_/P3HT/PEDOT:PSS/Au	14.9	670	58.0	5.8	[53]
	Sputtering	Mo/Sb_2_S_3_/CdS/ITO/Ag	12.5	696	33.3	2.9	[4]
	Sputtering	FTO/TiO_2_‐c/Sb_2_S_3_/P3HT/PEDOT:PSS/Au	12.9	587	60.2	4.6	This work

^a)^ FCA: Fast chemical approach;

^b)^ HT: Hydrothermal;

^c)^ CSS: closed space sublimation;

^d)^ RTE: rapid thermal evaporation;

^e)^ VTD: vapor transport deposition;

^f)^ ALD: atomic layer deposition.

Sputtering deposition lags far behind, with a reported top efficiency of only 2.9% to date (literature reports of sputtered Sb_2_S_3_ solar cells are listed in Table S1, Supporting Information). Therefore, the results of this report, with an efficiency of 4.6%, are promising but still require further investigation. The J_SC_ (currently below 13 mA cm^−2^) could be further improved by optimizing the quality of the absorber layer, allowing for a thicker Sb_2_S_3_ layer to be used. The optimum thickness of 120 nm in this work is insufficient. For instance, the Tang group found the optimum thickness to be ≈350 nm for the champion Sb_2_S_3_ solar cell (J_SC_ approaching 18 mA cm^−2^) using the hydrothermal method.^[^ ^48^ ^]^ Further optimization is necessary to enhance the V_OC_, which is inferior to that of high‐performance Sb_2_S_3_ solar cells. Notably, the processing of Sb_2_S_3_ films by sputtering has been underinvestigated compared to solution‐based methods, and there is a considerable scope for optimization.

### 2.3 Semitransparent Solar Cells

As discussed, RF magnetron sputtering allows for a controlled thin film growth rate, which means the device's transmittance can be predictable and tuned by varying the sputtering duration. Figure S21 (Supporting Information) shows the average visible transmittance (AVT) of the FTO/TiO_2_/Sb_2_S_3_ (argon‐sulfur annealed) stack. AVT progressively increased from 0.8% to 28.0% as the Sb_2_S_3_ layer thickness decreased from 250 to 40 nm.

**Figure 7:**
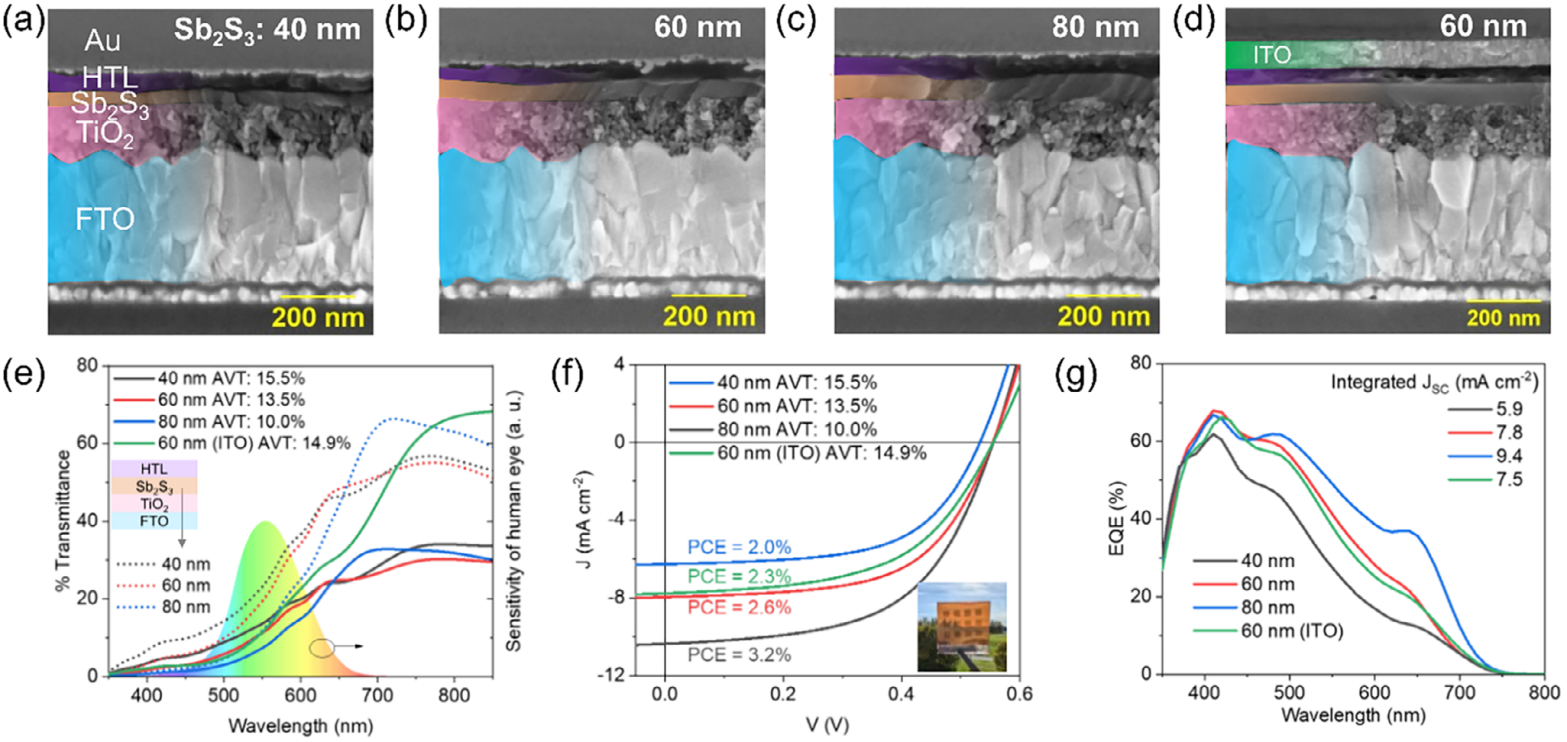
Cross‐section SEM images of semitransparent Sb_2_S_3_ solar cells with Sb_2_S_3_ absorber thickness of a) 40, b) 60, and c) 80 nm using ultrathin Au (≈10 nm) as transparent top electrodes. d) Semitransparent solar cell with 60 nm absorber using ITO as the transparent electrode. e) Transmittance curves for FTO/TiO_2_/Sb_2_S_3_/HTL (dotted lines) and complete semitransparent devices using ultrathin Au and ITO top electrodes (solid lines). f) *J–V* curves and g) EQE spectra of the devices with 40, 60, and 80 nm Sb_2_S_3_ absorber layers. An ITO‐based semitransparent solar cell with a 60 nm Sb_2_S_3_ absorber is also included for comparison in (f,g). A photograph of a typical ultrathin Au‐based semitransparent solar cell in (f). P3HT‐PEDOT:PSS was the HTL in these devices.

Semitransparent solar cells with varying thicknesses (AVT target: 15–20%) were fabricated, where the top Au contact was thinned down to ≈10 nm to allow sufficient transparency and conductivity, as reported in our previous work.^[^ ^13^ ^]^ Indium‐doped tin oxide (ITO) was also used to increase the overall transparency of the device. Transmittance spectra of Au (≈10 nm, AVT: 66.0%) and ITO (80–90 nm, AVT: 84.7%) are shown in Figure S22 (Supporting Information). The cross‐section SEM images of the semitransparent devices show a compact morphology of Sb_2_S_3_ layers even at these low thicknesses (<80 nm) (**Figure** **7**a–d). The AVTs (air as reference) of the complete devices using ultrathin Au electrodes are shown in Figure 7e. AVT for semitransparent devices increases from 10.0 to 13.5 to 15.5% for Sb_2_S_3_ thicknesses of 80, 60, and 40 nm, respectively. Similarly, the AVTs of ITO‐based semitransparent devices are shown in Figure S23 (Supporting Information). ITO devices showed an enhanced AVT of 14.9% using a 60 nm Sb_2_S_3_ absorber layer compared to that of 13.5% using ultrathin Au.

*J–V* curves of these semitransparent solar cells are shown in Figure 7f, and the device performance parameters and AVTs are listed in **Table** **3**. The EQE spectra are also shown in Figure 7g. The semitransparent devices (using 10 nm Au) show promising PCEs of 3.2, 2.6, and 2.0% for Sb_2_S_3_ thicknesses of 80, 60, and 40 nm, respectively. Light utilization efficiency (LUE = PCE × AVT) is a simple parameter used to compare the performance of semitransparent solar cells.^[^ ^54^ ^]^ A comparison table of LUEs of reported semitransparent Sb_2_S_3_ solar cells is given in Table S12 (Supporting Information). An LUE of 0.35 is obtained for using a 60 nm Sb_2_S_3_ layer, comparable to other prominent deposition methods.

**Table 3:** Device performance parameters of semitransparent Sb_2_S_3_ solar cell devices.

Transparent electrode	*d*_Sb2S3_ [nm]	*J*_SC_ [mA cm^−2^]	*V*_OC_ [mV]	FF [%]	PCE [%]	AVT [%]	LUE
Au (10 nm)	40	6.1 ± 0.1 (6.3)	530 ± 3 (532)	57.5 ± 1.1 (58.9)	1.9 ± 0.1 (2.0)	15.5	0.31
	60	7.8 ± 0.2 (8.0)	561 ± 14 (554)	57.6 ± 1.1 (58.8)	2.5 ± 0.1 (2.6)	13.5	0.35
	80	10.3 ± 0.1(10.4)	558 ± 9 (554)	54.2 ± 0.9 (55.8)	3.1 ± 0.1 (3.2)	10.0	0.32
ITO	40	6.9 ± 0.1 (6.9)	504 ± 10 (514)	50.0 ± 1.6 (51.4)	1.7 ± 0.1 (1.8)	16.8	0.30
	60	7.7 ± 0.1 (7.8)	553 ± 8 (554)	50.1 ± 2.4 (54.0)	2.1 ± 0.1 (2.3)	14.9	0.34
	80	9.2 ± 0.3 (9.3)	563 ± 11 (575)	48.0 ± 1.5 (50.0)	2.5 ± 0.1 (2.7)	11.8	0.32
CuSCN/ITO	60	8.1 ± 0.3 (8.4)	511 ± 17 (516)	46.8 ± 1.0 (47.0)	1.9 ± 0.1(2.0)	20.5	0.41

The average and standard deviation of the top eight cells for each condition are reported. The parameters of the champion devices are reported in parentheses. d_Sb2S3_ is the absorber thickness.

The ITO‐based semitransparent devices show slightly lower PCEs compared to Au, probably because of the sub‐optimum properties of ITO (such as energy level alignment with HTL, reactivity, etc.). The *J–V* curves and EQE spectra of the ITO‐based semitransparent devices (40, 60, and 80 nm absorbers) are shown in Figure S24a,b (Supporting Information), respectively. Nevertheless, the ITO electrode enhances the AVTs of the semitransparent devices as expected (Table 3).

**Figure 8:**
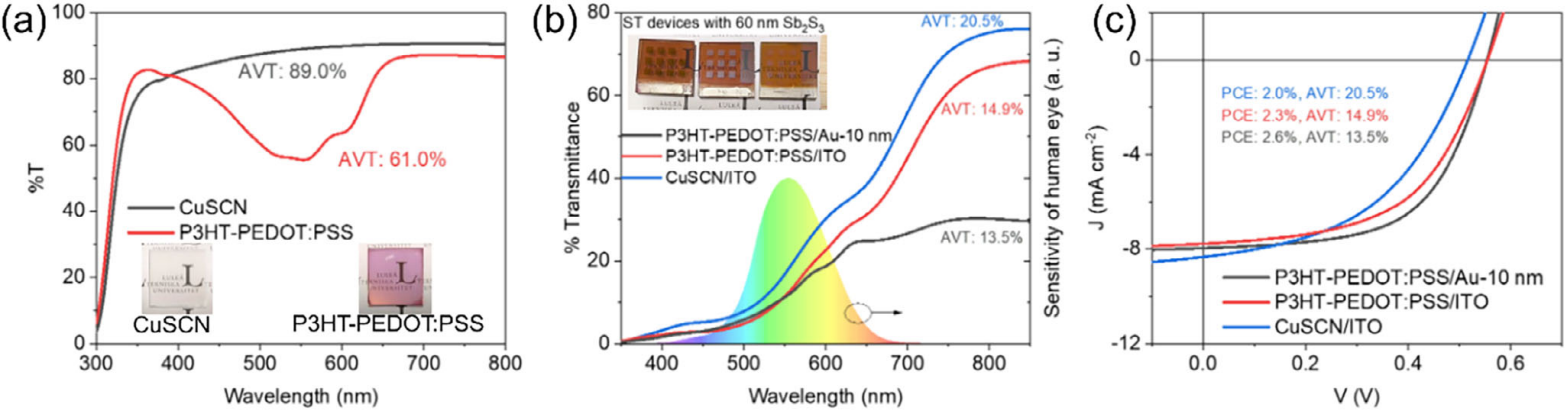
a) Transmittance curves of CuSCN and P3HT‐PEDOT:PSS HTLs. b) Transmittance curves of semitransparent solar cells using 60 nm Sb_2_S_3_ using CuSCN and P3HT‐PEDOT:PSS as HTLs. Both ultrathin Au and ITO‐based devices are shown for comparison. Photographs of the devices are also shown: left: P3HT‐PEDOT:PSS/Au‐10 nm, middle: P3HT‐PEDOT:PSS/Au‐10 nm, and right CuSCN/ITO. c) The *J–V* curves of semitransparent solar cell using CuSCN as HTL and ITO as the top transparent electrode. The curves for devices P3HT‐PEDOT:PSS as HTLs are shown for direct comparison.

Although promising results for semitransparent solar cells were obtained, the AVT is still less than 20%, even for the ITO‐based devices. This is primarily due to the low AVT of the P3HT as the HTL, which has a low E_g_ of 2.0 eV (thus a low AVT of 61%). To further improve the AVT, an inorganic, cost‐effective HTL, copper(I) thiocyanate (CuSCN), with a high AVT of 89% was used in place of P3HT. CuSCN was spin‐coated and dried as detailed in our previous studies.^[^ ^55^, ^56^ ^]^ The transmission curves of CuSCN and P3HT‐PEDOT:PSS films on glass are shown in **Figure** **8**a. Figure 8b,c presents a direct comparison of AVT and *J–V* curves of the semitransparent solar cells utilizing a 60 nm Sb_2_S_3_ absorber layer. The EQE curve of the CuSCN device is shown in Figure S25 (Supplementary Information) **Tak25**. By employing ITO as a transparent electrode (instead of Au‐10 nm) and CuSCN as an HTL (instead of P3HT‐PEDOT:PSS), an AVT of 20.5% was achieved. The CuSCN/ITO device yielded a PCE of 2.0% (with the highest LUE of 0.41). This is among the top LUEs (see Table S12, Supporting Information) with a potential for optimization at the CuSCN/ITO and Sb_2_S_3_/CuSCN interfaces. The origin of loss in PCE due to CuSCN as HTL instead of P3HT was reported in our previous study.^[^ ^56^ ^]^ Furthermore, the comprehensive optimization of ITO (conductivity vs transparency) was beyond the scope of this work. Nevertheless, the absence of an S‐kink (rollover in forward bias) in the *J–V* curve implies that the ITO electrode is compatible with the device structure and has a scope for improvement.^[^ ^57^ ^]^

The LUE value of 0.41 is promising for an emerging inorganic absorber, such as Sb_2_S_3_ and better than other emerging solar absorbers. However, it is still lower than established semitransparent inorganic solar cell technologies, such as a‐Si, CdTe, and CIGS, which have LUEs greater than 1.5.^[^ ^58^ ^]^ Organic and perovskite solar cells have traditionally had the highest LUEs among all semitransparent technologies (>5.0) but have inherent stability issues. Semitransparent sputtered Sb_2_S_3_ solar cells will also benefit from the rapid improvements in the PCE of opaque devices.

On the other hand, there are specific concerns that need to be studied urgently for these semitransparent solar cells to be applied as BIPV. These include: a) optimization of transparent electrodes, focusing on screening of alternatives to ITO like aluminum‐doped zinc oxide (AZO) and Indium‐zinc oxide (IZO); b) scaling study focusing on the redesigning of these electrodes to mitigate resistive losses during scaling, as these electrodes are generally less conductive than opaque electrodes. For example, a grid would be necessary to reduce resistive loss, albeit at the expense of some transparency^[^ ^59^ ^]^; c) optimization of wide bandgap inorganic HTL (CuSCN) to further improve the performance, since P3HT still outperforms CuSCN.^[^ ^56^ ^]^

## 3 Conclusion

In summary, we present a strategy to substantially improve the PCE in cadmium‐free superstrate structured semitransparent solar cells by obtaining a stoichiometric, highly compact, reproducible, and impurity‐free Sb_2_S_3_ film produced via RF magnetron sputtering. The post‐deposition annealing ambient was found to be the key processing parameter influencing the solar cell performance. A thorough optoelectronic characterization was conducted to examine the effects of annealing conditions on sputtered Sb_2_S_3_ thin films. A small amount of sulfur added during annealing modified the morphology and crystal orientation of the Sb_2_S_3_ film, leading to improved electron mobility and reduced defect‐related recombination in the solar cell device. Hence, the solar cell device using an argon‐sulfur annealed Sb_2_S_3_ absorber layer achieved a high PCE of 4.6%. In comparison, the device annealed without sulfur showed high resistance and significant recombination losses, resulting in a low PCE of 0.6%. The RF sputtering process produced compact ultrathin Sb_2_S_3_ films on bare TiO_2_, which was then utilized to fabricate semitransparent solar cells. These solar cells employed ultrathin Au (≈10 nm) as the top transparent electrode. The semitransparent solar cells achieved PCEs (AVTs) of 3.2% (10%), 2.6% (13.5%), and 2.0% (16.5%) for Sb_2_S_3_ layers of 80, 60, and 40 nm in thickness, respectively. Additionally, using ITO instead of Au (≈10 nm) as the top transparent electrode and CuSCN as the HTL (instead of P3HT) further enhanced the AVT to 20.5% (with PCE = 2.0% for 60 nm Sb_2_S_3_ absorber), resulting in one of the highest LUEs (0.41) for semitransparent Sb_2_S_3_ solar cells. This study demonstrates sputtering as an effective and scalable method for producing uniform, high‐quality, impurity‐free Sb_2_S_3_ films for high‐efficiency semitransparent solar cells.

## 4 Experimental Section

### Device Fabrication—Spin Coating of TiO_2_ Film as Electron Transport Layer

FTO substrates (TEC 15) were cleaned in an ultrasonic bath using Hellmanex III solution (2 vol.% in distilled water), distilled water, acetone, and isopropanol, respectively, for 10 min each. These films were dried at 120 °C for 10 min. A UV‐ozone treatment was done to increase the hydrophilicity of FTO using an ozone cleaner (HO‐TH‐UVT150, Holmarc, India). An electron transport layer, the TiO_2_ layer, was spin‐coated (in the ambient atmosphere) on the cleaned FTO using the sol‐gel method.^[^ ^55^ ^]^ In short, titanium (IV) isopropoxide (350 µL) was added to 5 mL of absolute ethanol (cold) in a 10 mL glass vial. To this solution, 65 µL diluted HCL (2 M diluted in absolute ethanol) was added dropwise. Vigorous magnetic stirring was done during the sol‐gel preparation, and the solution was kept stirring overnight. The resultant clear solution was filtered with a PTFE filter with a 0.45 µm pore size. The thickness of TiO_2_ was maintained at 120–130 nm by three spin coatings at 2000 rotations per minute (RPM). The thickness of the TiO_2_ film was not independently optimized but was based on the previous work.^[^ ^13^ ^]^ Films were dried at 200 °C for 10 min and cooled between the spin cycles. These films were then annealed at 500 °C for 50 min in a muffle furnace in air.

### Device Fabrication—RF Magnetron Sputtering of Sb_2_S_3_ Films

RF magnetron sputtering was done using a MiniLab 060 (by Moorfield nanotechnology, UK) with a 3‐inch Sb_2_S_3_ target (99.9% purity supplied by Advanced Engineering Materials Limited (AEM), China). Before sputtering, the sputtering chamber was evacuated below 5 × 10^−6^ mbar. The working pressure was maintained at 3 × 10^−3^ mbar by introducing 40 SCCM of high‐purity argon gas (99.99%) unless stated otherwise. RF power was set at 50 W. The FTO/TiO_2_ substrates were maintained at room temperature unless stated otherwise (the actual temperature of the temperature sensor varied between 25 and 30 °C). The thickness of the Sb_2_S_3_ films was tuned by varying the sputtering time.

### Device Fabrication—Post‐Deposition Treatment

Annealing was done in a tube furnace with two heating zones (Zone 1 and 2). The tube was first evacuated via a mechanical pump down to a 5 × 10^−2^ mbar pressure. During annealing, 200 SCCM argon (purity 99.9%) was introduced into the quartz tube to maintain the argon atmosphere of ≈1 mbar (using continuous pumping and adjusting the manual valve to control the vacuum). For argon‐only annealing (“Argon” devices for simplicity), both zones were kept at a fixed temperature of 320 °C. For sulfur vapor annealing in the argon‐sulfur atmosphere (“Argon‐sulfur” devices), a certain amount of sulfur powder (10, 25, 50, or 100 mg) was kept in zone 1 at 200 °C (unless stated otherwise). The substrates (FTO/TiO_2_/Sb_2_S_3_ (as deposited)) were placed in Zone 2 at varying temperatures of 300–380 °C. Annealing/sulfurization duration was 5–30 min. A schematic of the process is shown in the results section. The annealing parameters for argon‐sulfur annealed Sb_2_S_3_ films were optimized to obtain the best photovoltaic performance.

### Device Fabrication—HTL and Top Contact Deposition

P3HT‐PEDOT:PSS bilayer was employed as the hole transport/contact layer and prepared based on the previous reference.^[^ ^53^ ^]^ Briefly, P3HT (Ossila, M108) with a concentration of 10 mg mL^−1^ in chlorobenzene was spin‐coated (in an ambient atmosphere) at 2000 RPM. Then, a PEDOT:PSS solution (Ossila HTL Solar, without modification) was subsequently spin‐coated (in ambient atmosphere) on P3HT at 6000 RPM to improve the contact with Au. The P3HT‐PEDOT:PSS bilayer was then annealed in the tube furnace in an argon atmosphere at 120 °C for 10 min. CuSCN was deposited by spin coating at 4000 RPM from a 30 mg mL^−1^ solution in diethyl sulfide (stirred for more than 6 h at room temperature). The film was dried in air for 10 min at 90 °C.

Au top contacts (50–60 nm for opaque devices and ≈10 nm for semitransparent devices) were sputtered using a compact sputter coater Leica EM ACE200 (directional sputtering setting at RT with a working pressure of 4 × 10^−2^ mbar and base pressure of 2 × 10^−2^ mbar).^[^ ^56^ ^]^

Indium‐doped tin oxide (ITO) top transparent contact was deposited in the MiniLab 060 sputtering system with a 3‐inch ITO target (In_2_O_3_/SnO_2_ 90/10 wt.%, 99.9% purity supplied by AEM, China). Before sputtering, the sputtering chamber was evacuated below 5 × 10^−6^ mbar. The working pressure was maintained at 4 × 10^−3^ mbar by introducing 20 SCCM of high‐purity argon gas (99.99%) unless stated otherwise. RF power was set at 20 W (first 10 min) and 60 W (next 30 min). The FTO/TiO_2_/Sb_2_S_3_/HTL samples were mildly heated at 50 °C (the actual temperature of the temperature sensor varied between 50 and 55 °C). ITO deposition parameters were chosen to minimize the impact of sputtering damage to HTL based on previous reports.^[^ ^60^, ^61^, ^62^ ^]^ An extensive ITO contact optimization was beyond the scope of this work.

A laser‐cut metal mask defined the device's active area, which was 3 × 3 mm (9 mm^2^).

### Characterization and Measurements—UV–Vis‐NIR Transmittance

Transmittance characterizations were performed by a Cary 5000 spectrophotometer. The optical bandgap, E_g_, was extracted using Tauc's formula: (α*h*ν)^γ^ =  *C* (*h*ν − *E_g_*), where α is the absorption coefficient, *h*ν is the photon energy, *C* is a constant, and γ takes the values of 2 or ½ for direct and indirect bandgap, respectively.^[^ ^63^ ^]^ The absorption coefficient, α was calculated using the equation: α  =  *lnT*/*t*, where *T* is the transmittance and *t* is the thickness of the film (determined by cross‐section SEM).^[^ ^64^ ^]^ Average visible transmittance (AVT) was calculated using the equation^[^ ^54^, ^65^ ^]^:

(8) $$\mathrm{AVT}=\frac{\int T\left(\lambda \right)\cdot V\left(\lambda \right)\cdot \mathrm{AM}1.5G\left(\lambda \right)d\lambda }{\int V\left(\lambda \right)\cdot \mathrm{AM}1.5G\left(\lambda \right)d\lambda }$$

where *λ* is the wavelength, *T(λ)* is the transmission spectrum with air as a reference, *V(λ)* is the human eye's photopic response, and AM1.5G represents the standard solar photon flux.

### Characterization and Measurements—Scanning Electron Microscopy (SEM) and Energy Dispersive X‐Ray Spectroscopy (EDS)

Morphology and cross‐section of films/devices were characterized by Magellan 400 Field Emission Scanning Electron Microscopy (FESEM). Atomic ratios were obtained using the installed EDS detector (X‐Max 80 mm^2^ SDD, Oxford Instruments). Silicon substrates were used instead of FTO to avoid the EDS energy overlap of Sn and Sb. The characteristic X‐ray energies of Sn and Sb were very close (Sn Lβ is 3.662 keV and Sb Lα is 3.605 keV).^[^ ^62^ ^]^

### Characterization and Measurements—Grazing Incidence X‐Ray Diffraction (GIXRD)

GIXRD patterns at grazing incidence of 0.5° were collected on a PANalytical Aeris X‐ray diffraction diffractometer with a Cu K_α_ source and 1Der detector, operating at 40 kV, 15 mA.

### Characterization and Measurements—Atomic Force Microscopy (AFM)

The atomic force microscope (AFM) [NT‐MDT NTEGRA] was used to study the surface topography of the Sb_2_S_3_ thin films. The AFM topography imaging was performed in semi‐contact mode by using the Tap300Al‐G AFM probe (from BudgetSensors) having a nominal tip radius of ≈10 nm, resonant frequency of ≈300 kHz, and a force constant of ≈45 N m^−1^. All the AFM topography maps were recorded at a resolution of 256 × 256 pixels and a scan rate of 0.6 Hz. The obtained AFM data was then analyzed using Gwyddion software.

### Characterization and Measurements—Raman Measurements

The Raman spectra were acquired using a WiTec CRM‐200 Raman spectrometer. The laser with wavelength 532 nm was used to excite the samples through a 20×/0.4 Olympus objective. The data were analyzed using Witec Suit 4.0 software.

### Characterization and Measurements—X‐Ray Photoelectron Spectroscopy (XPS) and Ultraviolet Photoelectron Spectroscopy (UPS) Measurements

XPS and UPS measurements were carried out in a Nexsa G2 surface analysis system (Thermo Scientific) with a base pressure of ≈1 × 10^−9^ mbar. A brief (10 s) Ar^+^ ion sputtering was performed to remove any native carbon contamination prior to the XPS and UPS measurements. A monochromatic Al K‐alpha X‐ray source was used for XPS measurements. The XPS spectra were analyzed using the CasaXPS software. All XPS spectra were corrected for charging effects by calibration with adventitious carbon (C 1s C–C, 284.8 eV). A helium I excitation source (hν = 21.22 eV) was used for UPS measurements, and a bias of −5 V was applied to the sample surface to separate the low energy cutoff from the system response.

### Characterization and Measurements—High‐Resolution Transmission Electron Microscopy (HRTEM), Scanning Transmission Electron Microscopy (STEM), and Energy Dispersive X‐Ray Spectroscopy (EDS)

The TEM analysis was performed with a TALOS F200S G2 microscope with a Schottky field emitter operating at 200 keV. The instrument was also equipped with a dispersion micro‐analysis of energy (EDS) and the STEM accessory. STEM pictures were recorded using High‐Angle Annular Dark Field (HAADF) detectors. In this imaging mode, the intensity I was proportional to Z^1.7^t, where Z is the mean atomic number and t is the thickness of the specimen.

Sample preparation: The lamellae (from Si/TiO_2_/Sb_2_S_3_ stacks) were obtained using a Zeiss Crossbeam 340 equipped with a liquid metal source (Ga^+^); the surface of the sample was protected with 2 Pt layers for avoiding any gallium contamination. A 30 keV Ga+ beam ranging from 7 nA to 20 pA currents was used for the preliminary cuts, thinning, and rough polishing of the lamellae. The final polishing was completed with a 5 KeV/10 pA probe to ensure reduced amorphization of the surface.

SCAPS‐1D: the numerical simulation program, developed by Burgelman et al., was used to simulate the device energy band diagram at equilibrium.^[^ ^66^ ^]^ Details and parameters can be found in the previous work^[^ ^56^ ^]^ and Kondotas et al.^[^ ^67^ ^]^ The energy levels of Sb_2_S_3_ and TiO_2_ have been updated based on the results of this paper.

### Device Characterization

The current density‐voltage (*J–V*) curves of the solar cells were obtained using a Keithley 2400 source meter under simulated AM 1.5G irradiation (100 mW cm^−2^) with a standard 100 W Xe lamp‐based solar simulator Oriel LCS‐100. The illumination intensity was calibrated by a monocrystalline silicon reference cell (Oriel 91150 P/N). A metal mask with an aperture area of 4.91 mm^2^ (circle with a diameter of 2.5 mm) was used for photovoltaic performance measurements. For dark measurements, the active area of the device (3 × 3 mm = 9 mm^2^) was used. The external quantum efficiency (EQE) was measured using the Rera SpeQuest quantum efficiency system, calibrated by silicon solar cell reference.

## Conflict of Interest

The authors declare no conflict of interest.

## Author Contributions

P.K. contributed to conceptualization, methodology, investigation, writing – original draft, and formal analysis. J.P.T. contributed to investigation and formal analysis. P.K. contributed to investigation and formal analysis. S.Y. contributed to investigation, formal analysis, supervision, and review. A.G. contributed to investigation, formal analysis, supervision (TEM analysis and data processing), and review. N.G. contributed to investigation, formal analysis, supervision (TEM specimen preparation and TEM analysis), and review. K.T.L. supervised XPS/UPS measurements. V.M. supervised and reviewed the work. A.V. supervised, reviewed, and edited the manuscript. All authors approved the final version of the manuscript.

## Supporting information



Supporting Information

Supplemental Data File

## Data Availability

The data that support the findings of this study are available in the supplementary material of this article.
