# Supplementary material for: High‐Efficiency Semitransparent Solar Cells Based on Magnetron Sputtered Sb2S3 Thin Films
Source: Adv Sci (Weinh). 2025 Oct 17;13(2):e12103. doi: 10.1002/advs.202512103 (PMC12786377; doi:10.1002/advs.202512103)
Supplement: Supplementary file 1 — Supporting Information [file ADVS-13-e12103-s001.docx]

Supporting Information

**High-Efficiency Semitransparent Solar Cells Based on Magnetron Sputtered Sb_2_S_3_ Thin Films**

*Pankaj Kumar,^1*^ Pawan Kumar,^1^ Joseph P. Thomas,^2^ Alessandro Gradone,^3^ Nicola Gilli,^3^ Shujie You,^1^ Vittorio Morandi,^4^ Kam Tong Leung,^2^ Alberto Vomiero^1,5*^*

^1^Division of Materials Science, Department of Engineering Sciences and Mathematics, Luleå University of Technology, SE-971 87 Luleå, Sweden.

^2^WATLab and Department of Chemistry, University of Waterloo, Waterloo, Ontario, N2L 3G1, Canada

^3^Institute for Nanostructured Materials (ISMN) - CNR section of Bologna

Via Piero Gobetti 101, 40129, Bologna, Italy

^4^Institute for nanostructured materials (ISMN) - CNR, Strada Provinciale 35 d, n. 9, 00010 Montelibretti (RM), Italy

^5^Department of Molecular Sciences and Nanosystems, Ca' Foscari University of Venice, Via Torino 155, 30172, Venezia Mestre, Italy.

*Corresponding authors

Email: [pankaj.kumar@associated.ltu.se](mailto:pankaj.kumar@associated.ltu.se), [alberto.vomiero@ltu.se](mailto:alberto.vomiero@ltu.se)

**Table S1**. Comparison table for Sb_2_S_3_ solar cells using sputtering deposition.

| Device structure | Sputtering  Target^a^ | *V*_OC_  (V) | *J*_SC_  (mA cm^-2^) | FF  (%) | PCE (%) | Year | Ref |
| --- | --- | --- | --- | --- | --- | --- | --- |
| Mo/Sb_2_S_3_/CdS/ITO/Ag | Sb | 696 | 12.48 | 33.28 | 2.89 | 2024 | ^[1]^ |
| FTO/CdS/Sb_2_S_3_/Ag | Sb | 174 | 0.5 | ~25 | <0.1 | 2024 | ^[2]^ |
| Mo/Sb_2_S_3_/CdS/ITO/Ag | Sb_2_S_3_ | 442 | 3.58 | 30.89 | 0.49 | 2020 | ^[3]^ |
| FTO/SnO_2_/Sb_2_S_3_/Spiro-OMeTAD/Ag | Sb_2_S_3_ | 440 | 4.77 | 41.08 | 0.86 | 2019 | ^[4]^ |
| FTO/Sb_2_S_3_/CdS/ZnO/ITO/Al | Sb_2_S_3_ | 538 | 10.54 | 42.58 | 2.41 | 2019 | ^[5]^ |
| ^a^Sb target refers to Sb metal deposition followed by sulfurization. | | | | | | | |


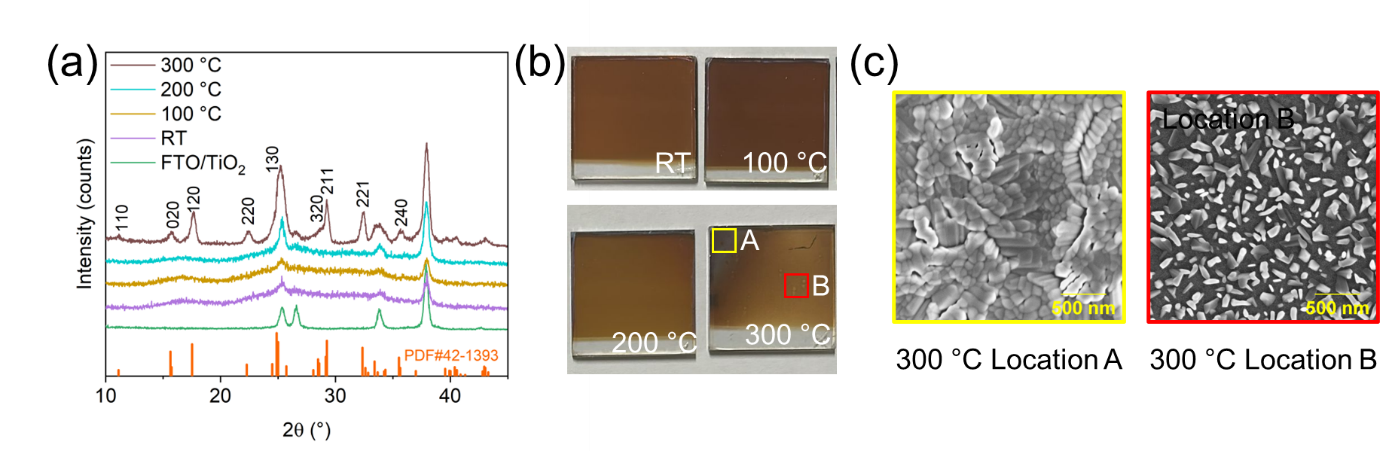


**Figure S1.** (a) XRD patterns of as-deposited thin films with varying substrate temperature during the deposition. (b) Photographs of as-deposited Sb_2_S_3_ films at substrate temperatures of RT, 100°C, 200°C, and 300°C. (c) SEM images showing two different regions of non-uniformity at a deposition temperature of 300 °C. Deposition at 300 °C results in a crystalline phase but incomplete coverage with large voids, which are unsuitable for solar cells.


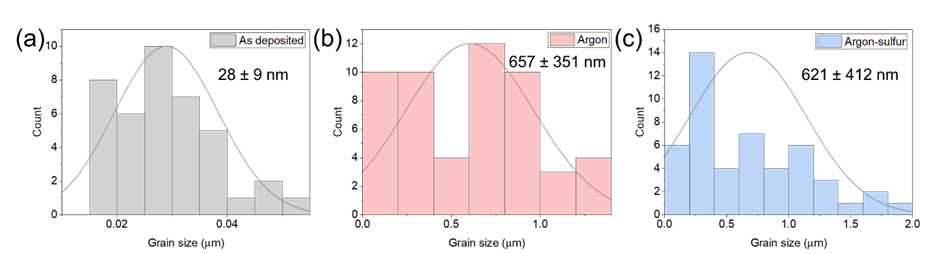


**Figure S2**. Grain size distribution of (a) As deposited, (b) Argon annealed at 320 °C, and (c) Argon-sulfur annealed at 320 °C.


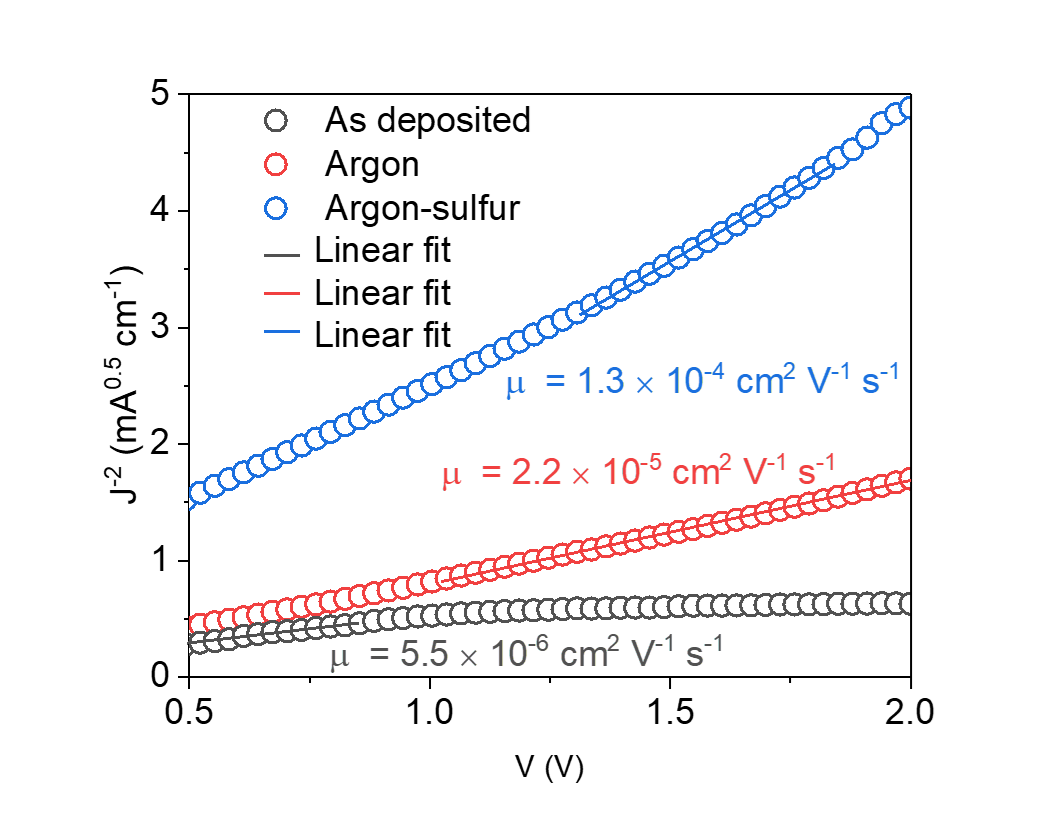


**Figure S3**. SCLC mobility calculation using Mott-Gurney law for electron-only SCLC devices.


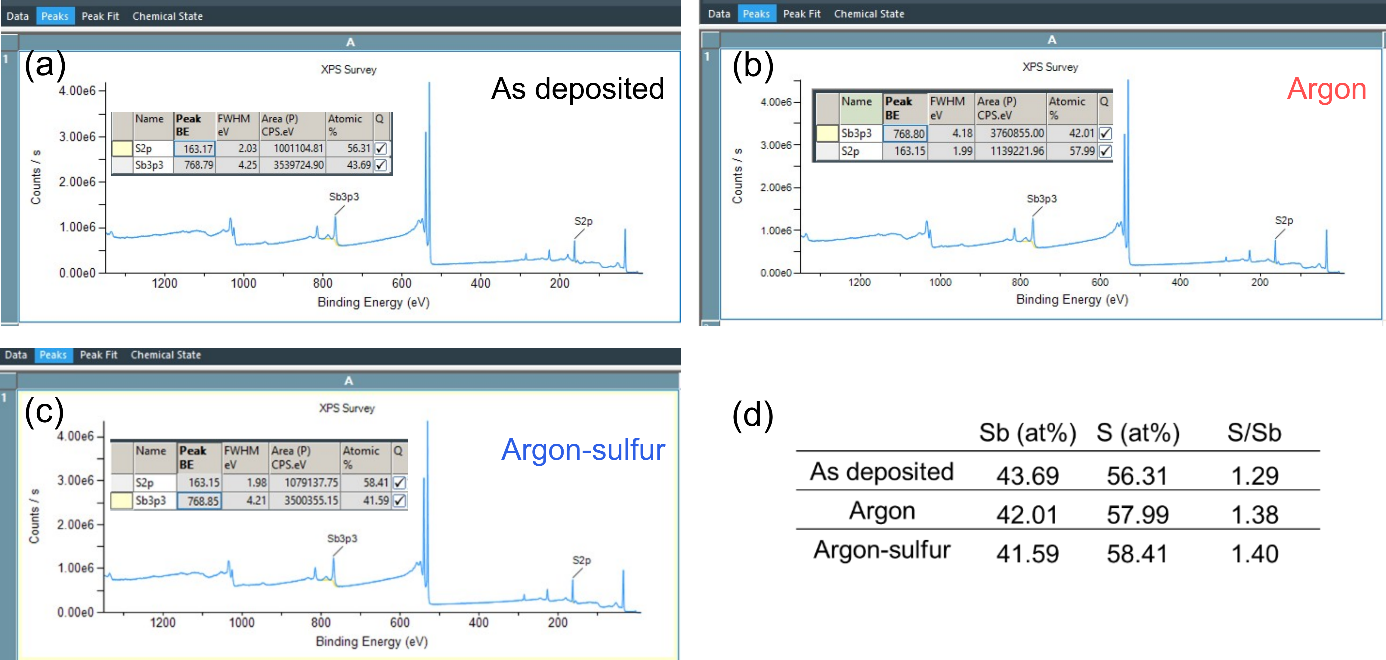


**Figure S4**. Atomic percentage of Sb and S obtained from the XPS survey spectra. Sb 3p_3/2_ and S2p regions were considered to avoid possible overlap with O1s and Sb3d_5/2_.

**Table S2**. Calculated energy levels from UPS and Eg values (as described in the main text).

| Film | E_C_ (ev) | E_V_ (ev) | E_F_ (eV) |
| --- | --- | --- | --- |
| Sb_2_S_3_-argon | -3.04 | -5.14 | -4.24 |
| Sb_2_S_3_-argon | -3.47 | -5.18 | -4.23 |
| Sb_2_S_3_-argon-sulfur | -3.49 | -5.22 | -4.22 |
| TiO_2_ | -4.20 | -7.62 | -4.52 |


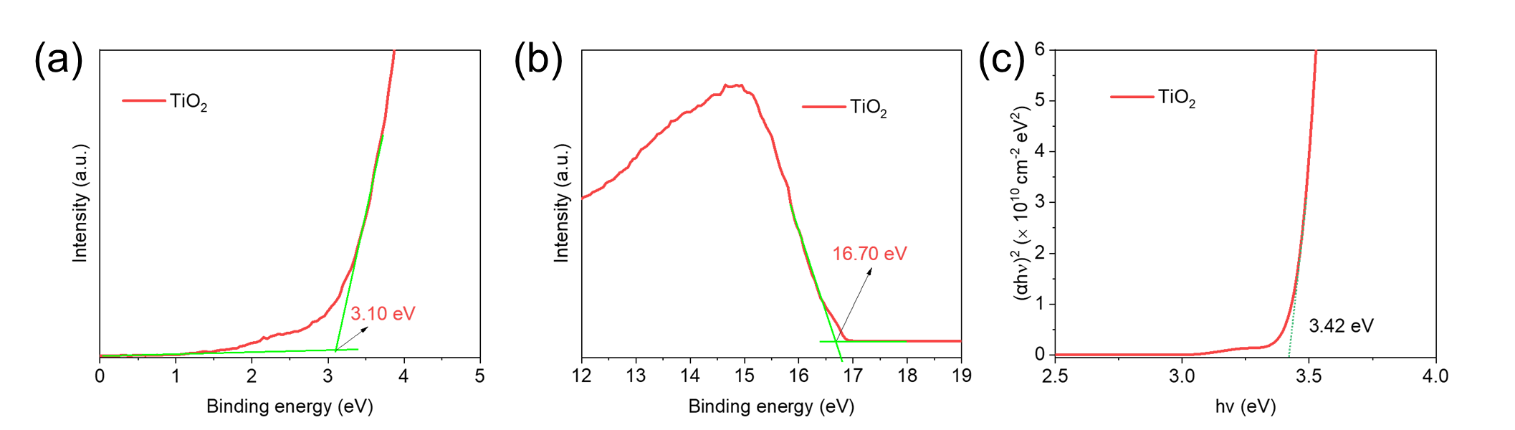


**Figure S5**. Ultraviolet photoelectron spectroscopy (UPS) spectra of TiO_2_ thin film. (a) valence band region and (b) secondary electron cut-off region. (c) The bandgap of TiO_2_ (3.42 eV) was calculated using a Tauc plot.


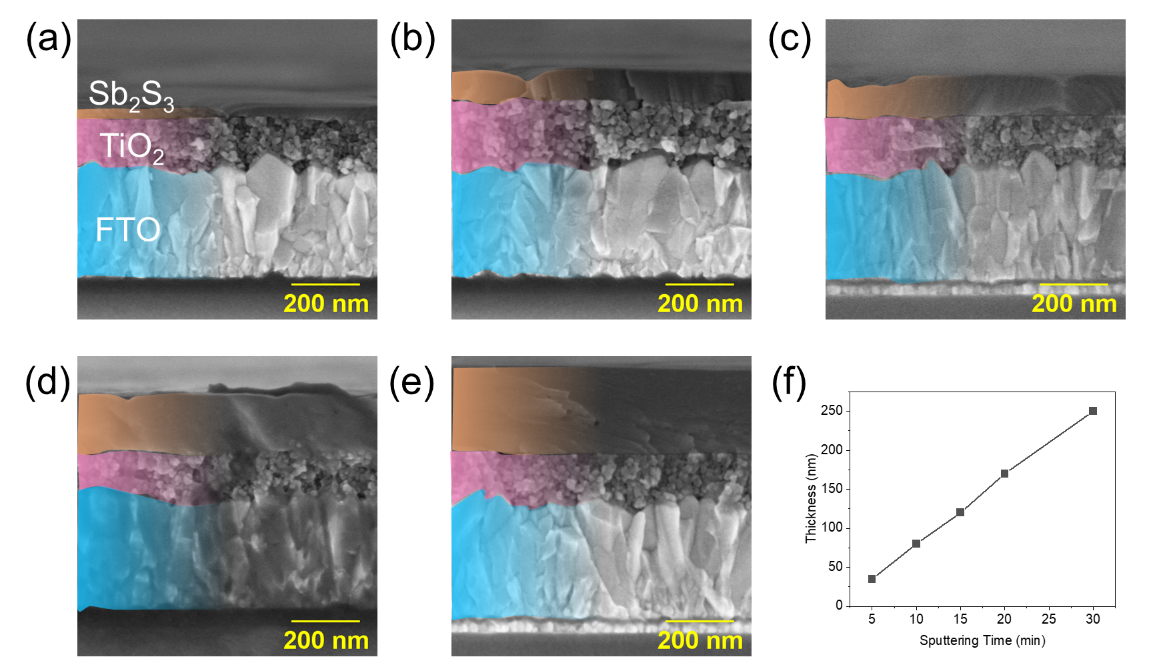


**Figure S6**. Thickness variation with sputtering times. (a) 5 min (b) 10 min, (c) 15 min, (d) 20 min, and (e) 30 min. (d) Plot of sputtering vs. thickness of Sb_2_S_3_ absorber. These films were post-annealed in an argon-sulfur atmosphere at 320 °C for 15 min.


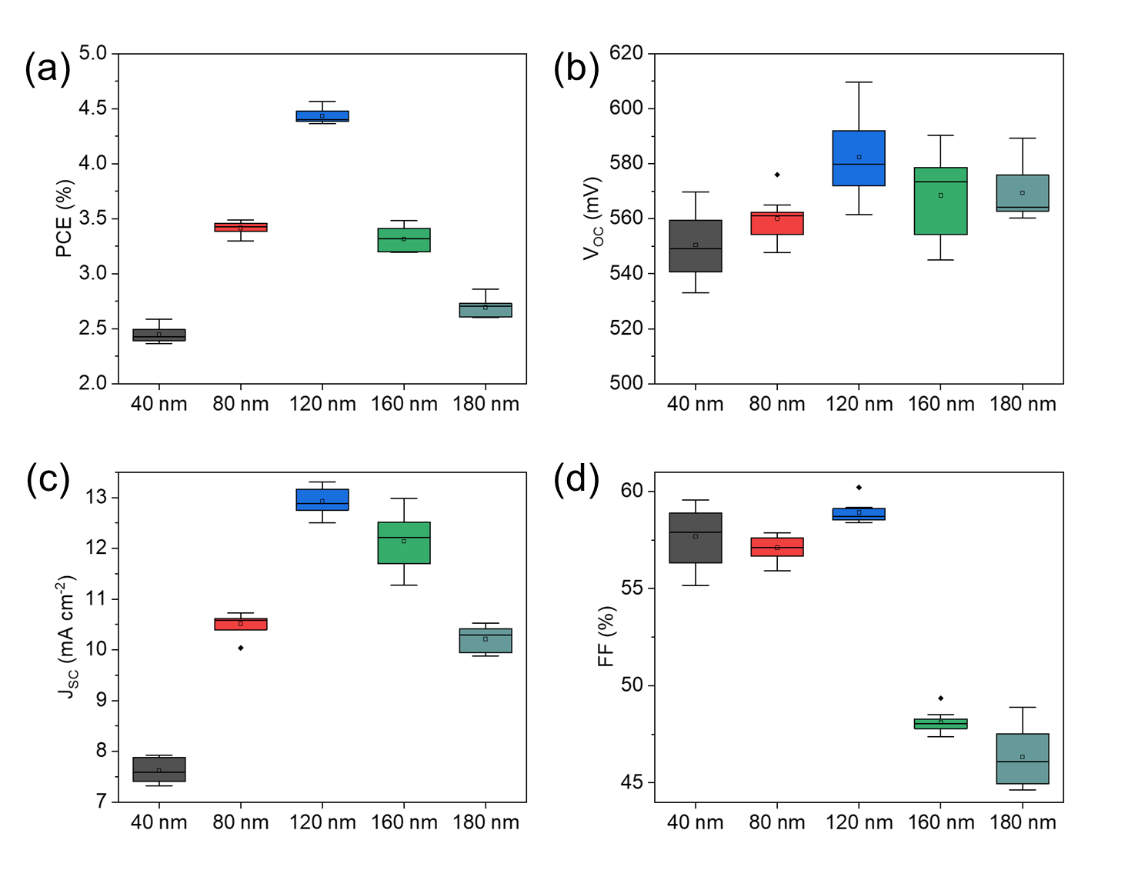


**Figure S7**. Statistical boxplots of photovoltaic parameters for Sb_2_S_3_ solar cells based on Sb_2_S_3_ film annealed in 25 mg Sulfur (at 200 °C in Zone 1) with varying thicknesses of the Sb_2_S_3_ absorber: (a) PCE, (b) V_OC_, (c) J_SC_, and (d) FF.

**Table S3**. Comparison of photovoltaic parameters for Sb_2_S_3_ solar cells based on Sb_2_S_3_ film annealed in 25 mg Sulfur (at 200 °C in Zone 1) with varying thicknesses of the Sb_2_S_3_ absorber

| Thickness | J_SC_ [mA cm^-2^] | V_OC_ [mV] | FF [%] | PCE [%] |
| --- | --- | --- | --- | --- |
| 40 nm | 7.9 (7.7 ± 0.2) | 568 (553 ± 10) | 57.9 (57.6 ± 9.9) | 2.6 (2.4 ± 0.1) |
| 80 nm | 10.7 (10.6 ± 0.1) | 562 (563 ± 6) | 57.9 (57 .3 ± 0.4) | 3.5 (3.4 ± 0.1) |
| 120 nm | 12.9 (12.9 ± 0.3) | 587 (582 ± 14) | 60.2 (58.9 ± 0.5) | 4.6 (4.4 ± 0.1) |
| 160 nm | 12.4 (12.1 ± 0.5) | 590 (570 ± 15) | 47.7 (48.1 ± 0.6) | 3.5 (3.3 ± 0.1) |
| 180 nm | 9.9 (10.2 ± 0.2) | 589 (569 ± 9) | 48.9 (46.3 ± 1.4) | 2.9 (2.7 ± 0.1) |
| ^a^Mean and standard deviation (SD), given in brackets, were calculated from the top eight devices. | | | | |


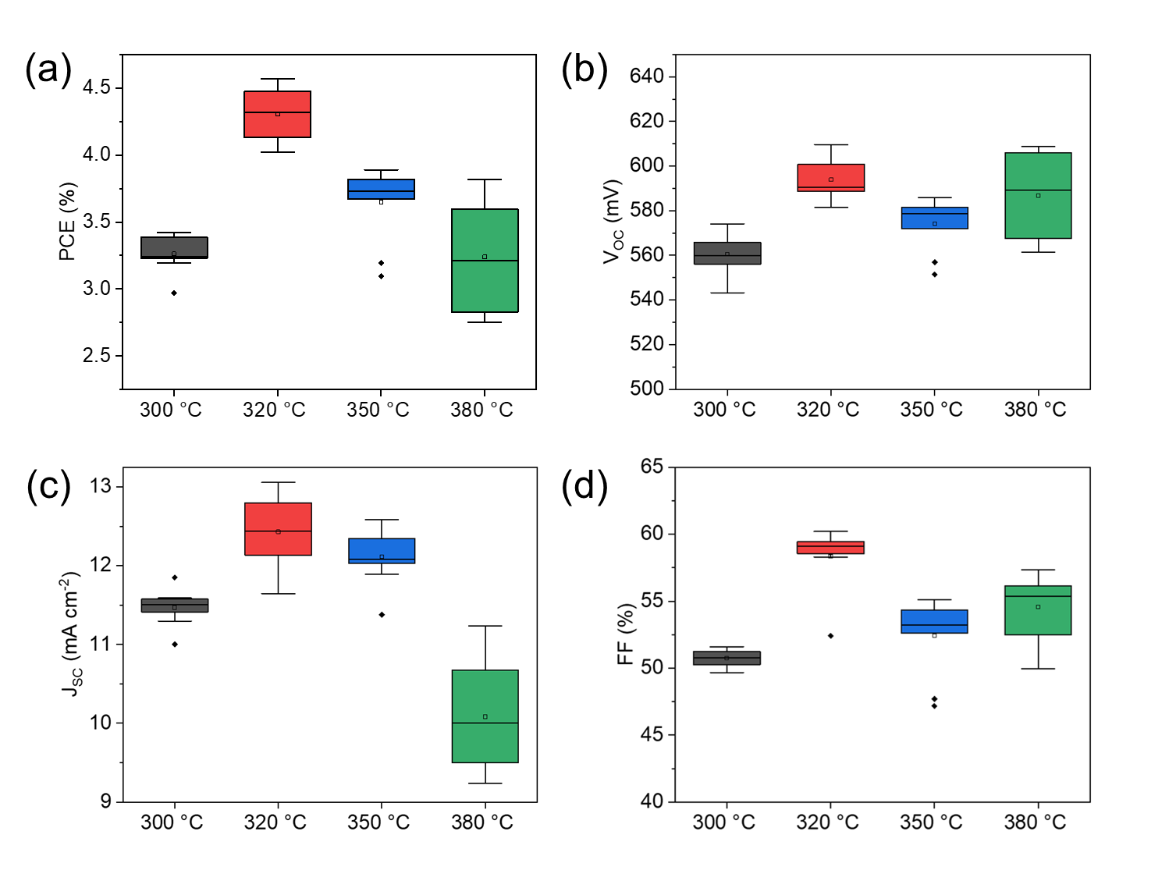


**Figure S8**. Statistical boxplots of photovoltaic parameters for Sb_2_S_3_ solar cells based on Sb_2_S_3_ film annealed with 25 mg Sulfur (at 200 °C in Zone 1) while varying annealing (sulfurization) temperatures of the FTO/TiO_2_/Sb_2_S_3_ samples (kept in Zone 2): (a) PCE, (b) V_OC_, (c) J_SC_, and (d) FF.

**Table S4**. Comparison of photovoltaic parameters for Sb_2_S_3_ solar cells based on Sb_2_S_3_ film annealed with 25 mg Sulfur (at 200 °C in Zone 1) while varying annealing (sulfurization) temperatures of the FTO/TiO_2_/Sb_2_S_3_ samples (kept in Zone 2).

| Annealing temperature [Zone 2](°C) | J_SC_ [mA cm^-2^] | V_OC_ [mV] | FF [%] | PCE [%] |
| --- | --- | --- | --- | --- |
| 300 | 11.8 (11.5 ± 0.1) | 563 (563 ± 7) | 51.3 (51.0 ± 0.5) | 3.4 (3.3 ± 0.1) |
| 320 | 12.9 (12.4 ± 0.4) | 587 (594 ± 9) | 60.2 (58.3 ± 2.3) | 4.6 (4.3 ± 0.2) |
| 350 | 12.6 (12.2 ± 0.4) | 582 (579 ± 4) | 53.1 (53.7 ± 0.8) | 3.9 (3.8 ± 0.1) |
| 380 | 11.6 (10.3 ± 0.6) | 597 (592 ± 15) | 56.9 (55.2 ± 2.2) | 3.8 (3.4 ± 0.3) |
| ^a^Mean and standard deviation (SD), given in brackets, were calculated from the top eight devices. | | | | |


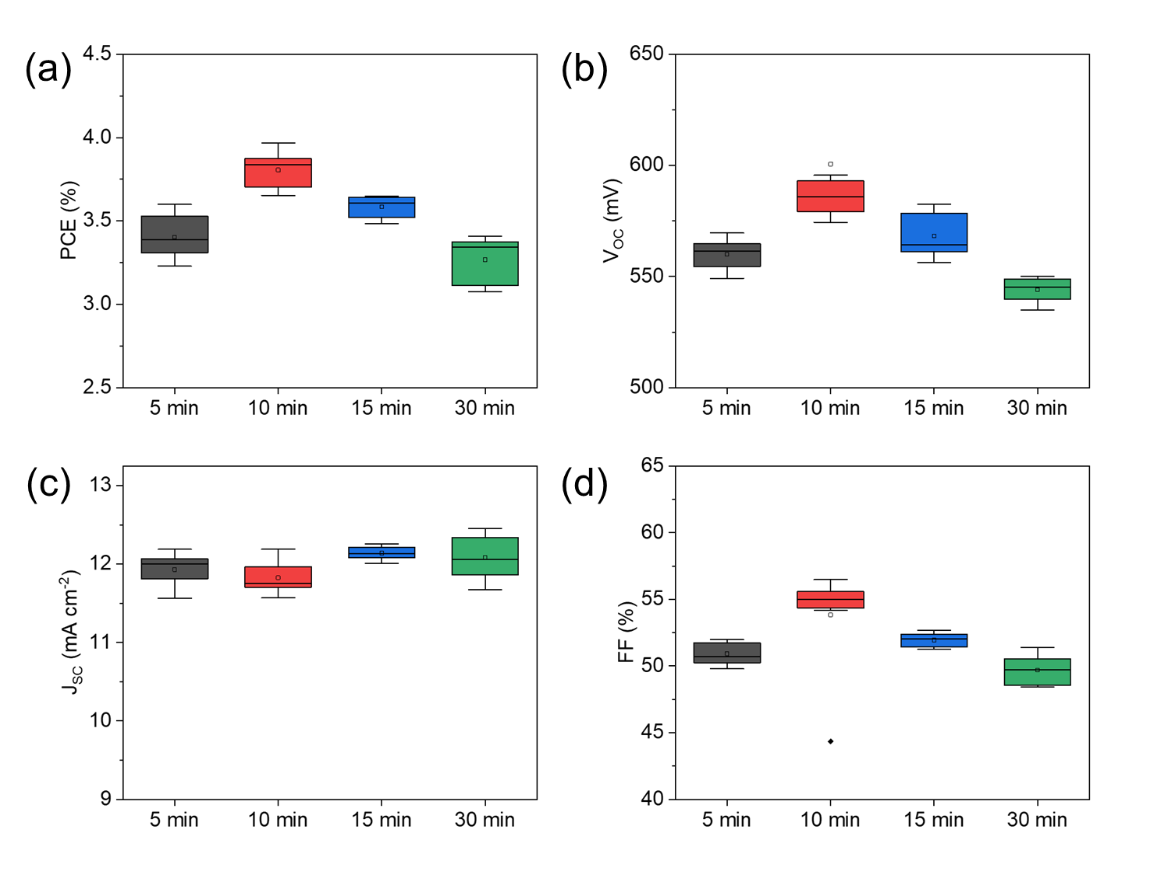


**Figure S9**. Statistical boxplots of photovoltaic parameters for Sb_2_S_3_ solar cells based on Sb_2_S_3_ film annealed with 25 mg Sulfur (Zone 1 at 200 °C) while varying annealing (sulfurization) duration keeping the FTO/TiO_2_/Sb_2_S_3_ samples (Zone 2) at 320 °C : (a) PCE, (b) V_OC_, (c) J_SC_, and (d) FF.

**Table S5**. Comparison of photovoltaic parameters for Sb_2_S_3_ solar cells based on Sb_2_S_3_ film annealed with 25 mg Sulfur (Zone 1 at 200 °C) while varying annealing (sulfurization) duration, keeping the FTO/TiO_2_/Sb_2_S_3_ samples (Zone 2) at 320 °C.

| Annealing duration [min] | J_SC_ [mA cm^-2^] | V_OC_ [mV] | FF [%] | PCE [%] |
| --- | --- | --- | --- | --- |
| 5 | 12.2 (11.9 ± 0.2) | 570 (561 ± 6) | 51.9 (51.1 ± 0.8) | 3.6 (3.4 ± 0.1) |
| 10 | 11.9(11.8 ± 0.2) | 589 (601 ± 43) | 56.5 (53.8 ± 3.6) | 4.0 (3.8 ± 0.1) |
| 15 | 12.3 (12.1 ± 0.1) | 565 ( 568 ± 9) | 52.7 (51.9 ± 0.5) | 3.6 (3.6 ± 0.1) |
| 30 | 12.3 (12.1 ± 0.3) | 548 (544 ± 5) | 50.4 (49.7 ± 1.0) | 3.4 (3.3 ± 0.1) |
| ^a^Mean and standard deviation (SD), given in brackets, were calculated from the top eight devices. | | | | |


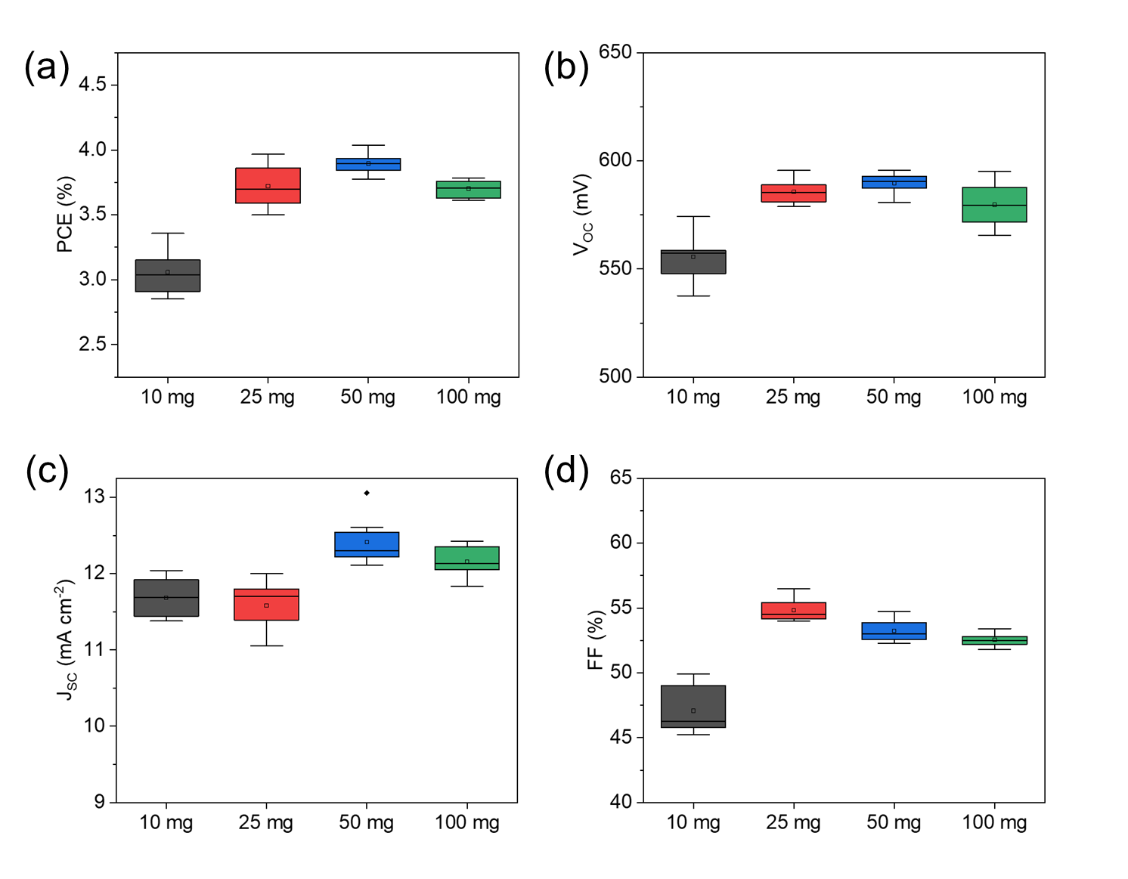


**Figure S10**. Statistical boxplots of photovoltaic parameters for Sb_2_S_3_ solar cells based on Sb_2_S_3_ films annealed in the argon-sulfur atmosphere with varying amounts of sulfur (at 200 °C in Zone 1): (a) PCE, (b) V_OC_, (c) J_SC_, and (d) FF. FTO/TiO_2_/Sb_2_S_3_ samples were kept at 320°C.

**Table S6**. Comparison of photovoltaic parameters for Sb_2_S_3_ solar cells based on Sb_2_S_3_ films annealed in the argon-sulfur atmosphere with varying amounts of sulfur (at 200 °C in Zone 1).

| Sulfur amounts [mg] | J_SC_ [mA cm^-2^] | V_OC_ [mV] | FF [%] | PCE [%] |
| --- | --- | --- | --- | --- |
| 10 | 11.9 (11.8 ± 0.2) | 574 (557 ± 11) | 49.1 (47.5 ± 1.5) | 3.4 (3.1 ± 0.2) |
| 25 | 11.9 (11.6 ± 0.3) | 589 (586 ± 6) | 56.5 (54.9 ± 0.8) | 4.0 (3.7 ± 0.1) |
| 50 | 13.1 (12.4 ± 0.3) | 590 (590 ± 4) | 52.4 (53.2 ± 0.8) | 4.0 (3.9 ± 0.1) |
| 100 | 12.4 (12.2 ± 0.2) | 581 (582 ± 8) | 52.4 (52.6 ± 0.5) | 3.8 (3.7 ± 0.0) |
| ^a^Mean and standard deviation (SD), given in brackets, were calculated from the top eight devices. | | | | |


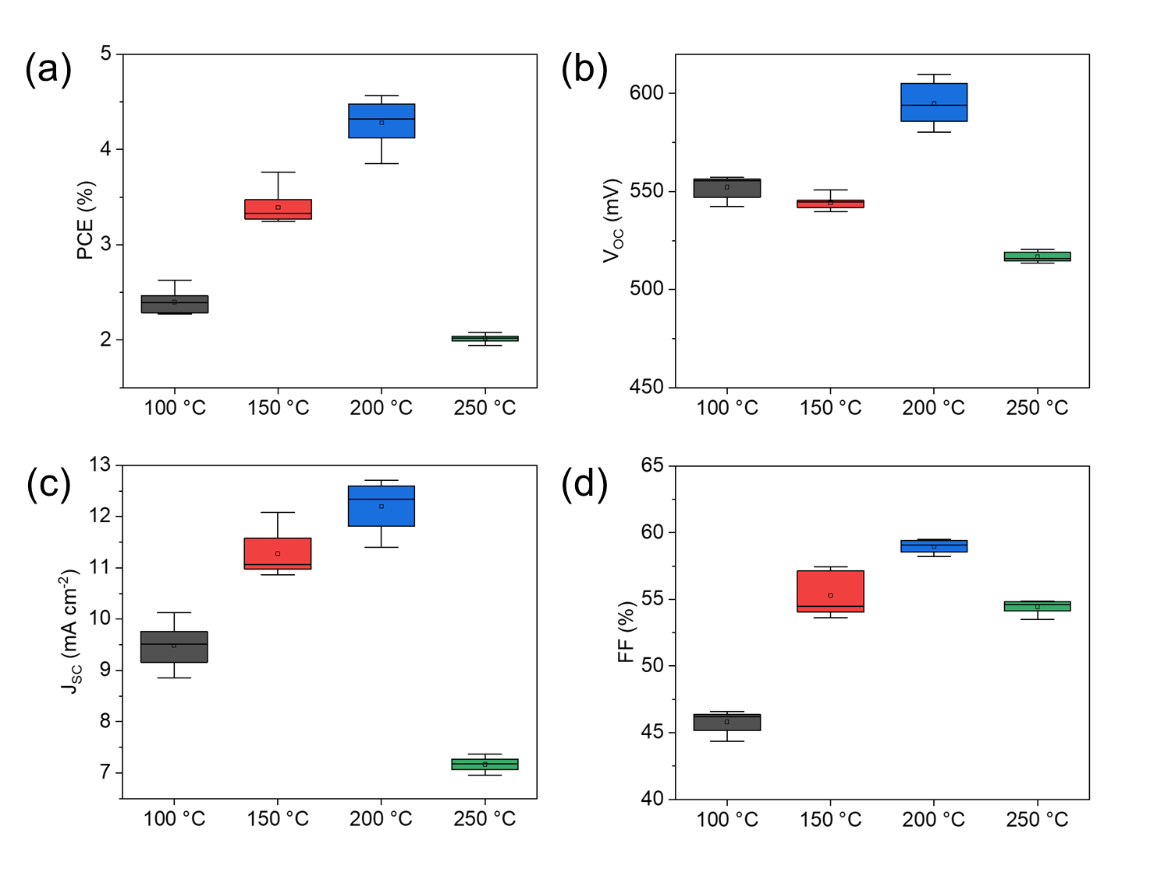


**Figure S11**. Statistical boxplots of photovoltaic parameters for Sb_2_S_3_ solar cells based on FTO/TiO_2_/Sb_2_S_3_ samples at 320 °C (in Zone 2) while varying Zone 1 (with 25 mg sulfur) temperature: (a) PCE, (b) V_OC_, (c) J_SC_, and (d) FF.

**Table S7**. Comparison of photovoltaic parameters for Sb_2_S_3_ solar cells based on FTO/TiO_2_/Sb_2_S_3_ samples at 320 °C (in Zone 2) while varying Zone 1 (with 25 mg sulfur) temperature.

| Sulfur temperatue (zone 1) | J_SC_ [mA cm^-2^] | V_OC_ [mV] | FF [%] | PCE [%] |
| --- | --- | --- | --- | --- |
| 100 °C | 10.1 (9.5 ± 0.4) | 557 (552 ± 5) | 46.6 (45.8 ± 0.8) | 2.6 (2.4 ± 0.1) |
| 150 °C | 12.1(11.3 ± 0.4) | 544 (544 ± 3) | 57.3 (55.3 ± 1.5) | 3.8 (3.4 ± 0.2) |
| 200 °C | 12.7 (12.2 ± 0.4) | 605 (595 ± 10) | 59.4 (59.0 ± 0.5) | 4.6 (4.3 ± 0.2) |
| 250 °C | 7.4 (7.2 ± 0.1) | 515 (517 ± 2) | 54.9 (54.4 ± 0.5) | 2.1 (2.0 ± 0.0) |
| ^a^Mean and standard deviation (SD), given in brackets, were calculated from the top eight devices. | | | | |


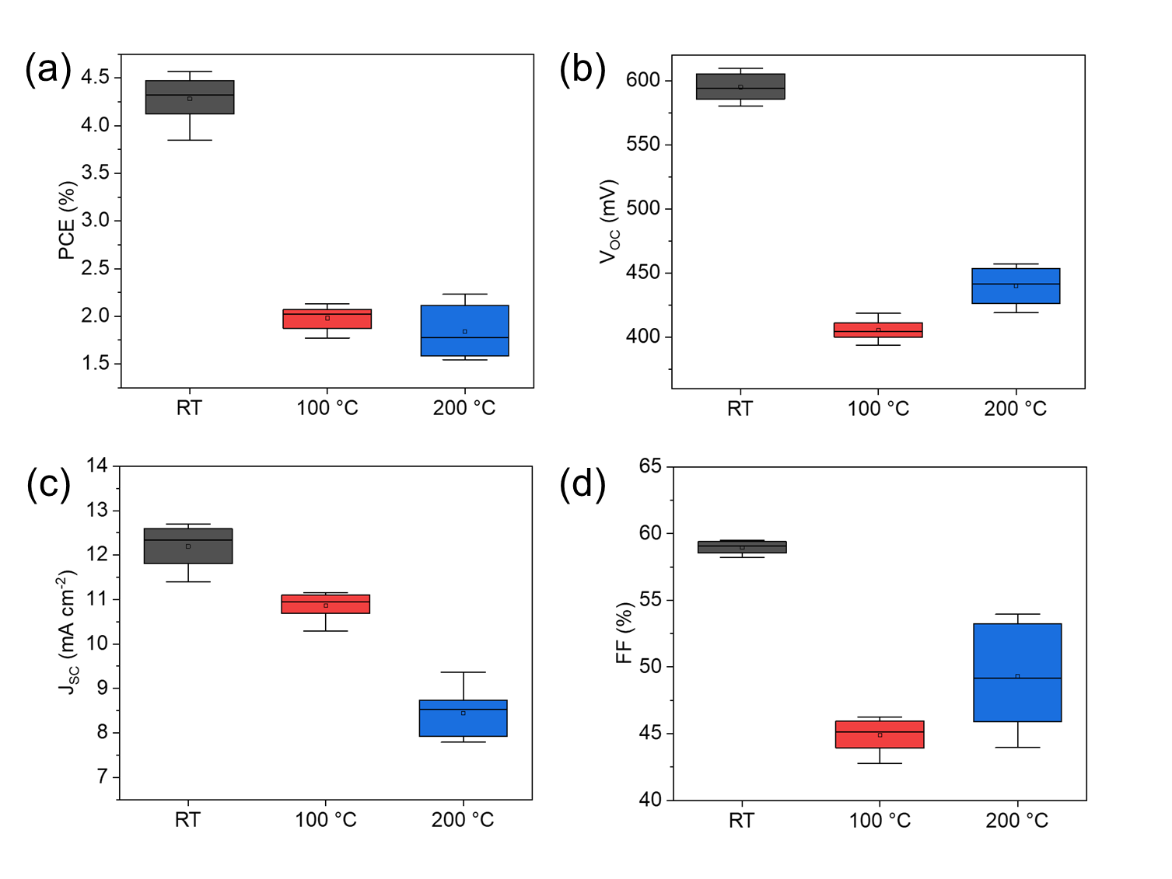


**Figure S12**. Statistical boxplots of photovoltaic parameters for Sb_2_S_3_ solar cells varying sputtering temperatures: (a) PCE, (b) V_OC_, (c) J_SC_, and (d) FF. **Note**: The deposition rates were lower for 100°C and 200 °C (suggesting material loss), so the deposition duration was increased to obtain ~120 nm thickness to compare with the RT deposited devices.

**Table S8**. Comparison of photovoltaic parameters for Sb_2_S_3_ solar cells varying sputtering temperatures. The deposition rates were lower for 100°C and 200 °C (suggesting material loss), so the deposition duration was increased to obtain ~120 nm thickness to compare with the RT deposited devices.

| Substrate temperature during sputtering | J_SC_ [mA cm^-2^] | V_OC_ [mV] | FF [%] | PCE [%] |
| --- | --- | --- | --- | --- |
| RT (25 °C) | 12.7 (12.2 ± 0.4) | 605 (595 ± 10) | 59.4 (59.0 ± 0.5) | 4.6 (4.3 ± 0.2) |
| 100 °C | 11.1 (10.9 ± 0.3) | 419 (405 ± 8) | 46.0 (44.9 ± 1.2) | 2.1 (2.0 ± 0.1) |
| 200 °C | 9.4 (8.4 ± 0.5) | 457 (440 ± 14) | 52.1 (49.3 ± 3.9) | 2.2 (1.8 ± 0.3) |
| ^a^Mean and standard deviation (SD), given in brackets, were calculated from the top eight devices. | | | | |


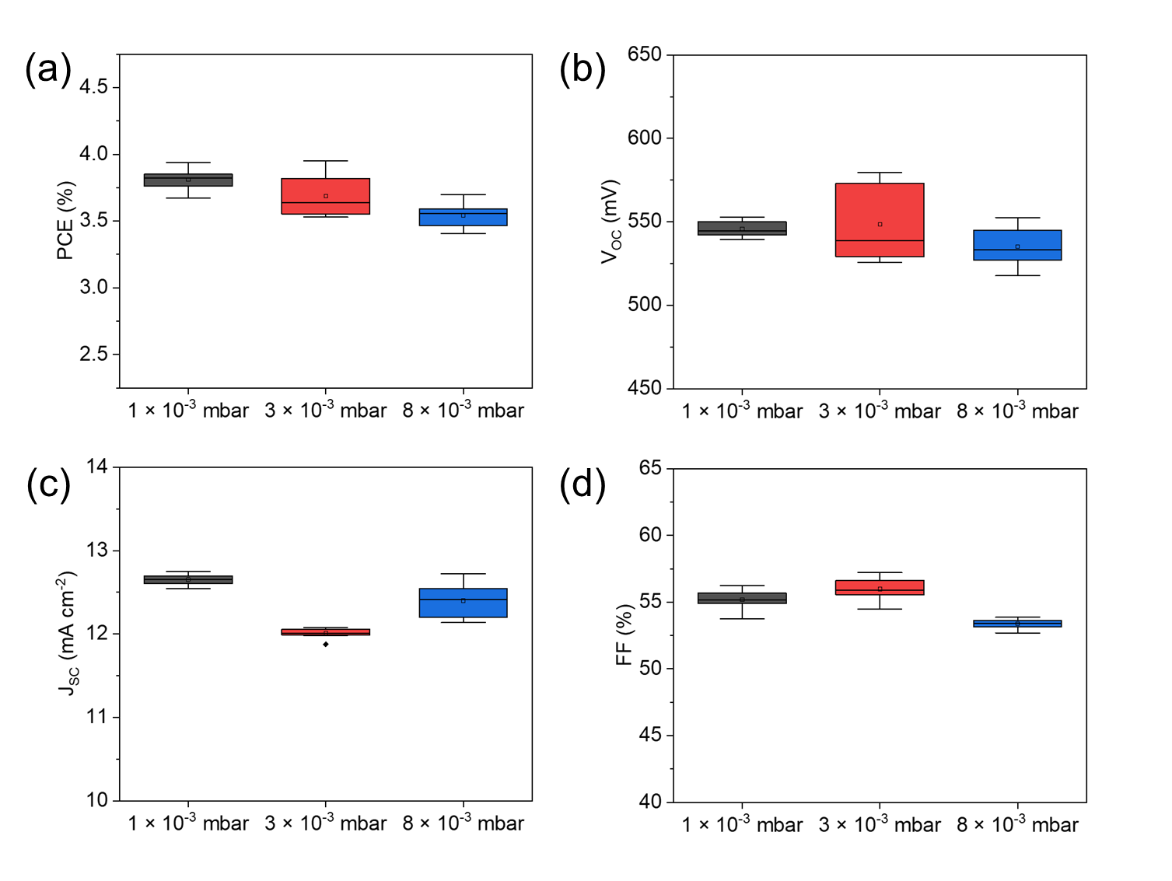


**Figure S13**. Statistical boxplots of photovoltaic parameters for Sb_2_S_3_ solar cells varying sputtering pressures while keeping the thickness of Sb_2_S_3_ at 120 nm: (a) PCE, (b) V_OC_, (c) J_SC_, and (d) FF. **Note**: The deposition rates were lower for the higher working pressure of 8 × 10^-3^ mbar, so the deposition duration was increased (25 min vs 15 min) to obtain ~120 nm thickness to compare with the lower pressures.

**Table S9**. Comparison of photovoltaic parameters for Sb_2_S_3_ solar cells varying sputtering pressures while keeping the thickness of Sb_2_S_3_ at 120 nm. **Note**: The deposition rates were lower for the higher working pressure of 8 × 10^-3^ mbar, so the deposition duration was increased (25 min vs. 15 min for lower deposition pressures) to obtain ~120 nm thickness to compare with the lower pressures.

| Working pressure during sputtering [mbar] | J_SC_ [mA cm^-2^] | V_OC_ [mV] | FF [%] | PCE [%] |
| --- | --- | --- | --- | --- |
| 1 × 10^−3^ | 12.7 (12.7 ± 0.1) | 551 (546 ± 5) | 56.2 (55.2 ± 0.7) | 3.9 (3.8 ± 0.1) |
| 3 × 10^−3^ | 12.0 (12.0 ± 0.1) | 579 (548 ± 22) | 56.9 (56.0 ± 0.8) | 4.0 (3.7 ± 0.2) |
| 8 × 10^−3^ | 12.5 (12.4 ± 0.2) | 553 (535 ± 11) | 53.6 (53.4 ± 0.3) | 3.7 (3.5 ± 0.1) |
| ^a^Mean and standard deviation (SD), given in brackets, were calculated from the top eight devices. | | | | |


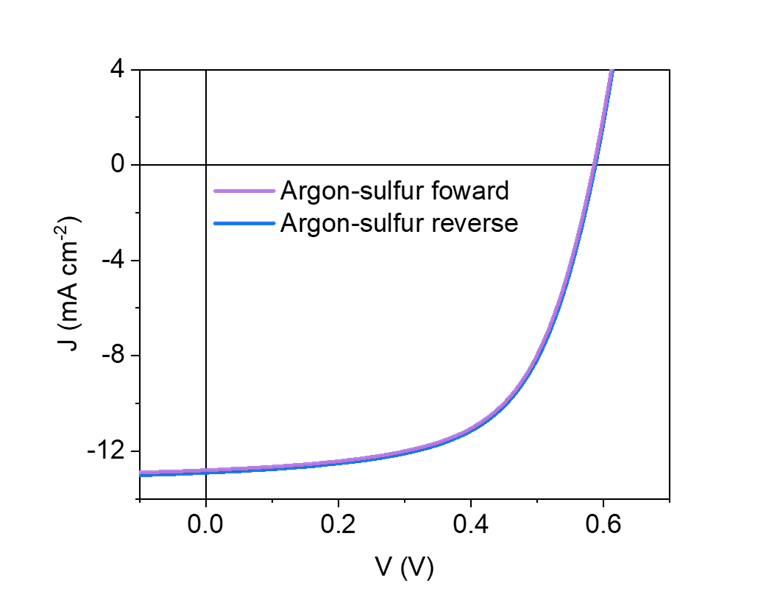


**Figure S14**. Forward and reverse *J*-*V* curves for a typical argon-sulfur annealed solar cell.


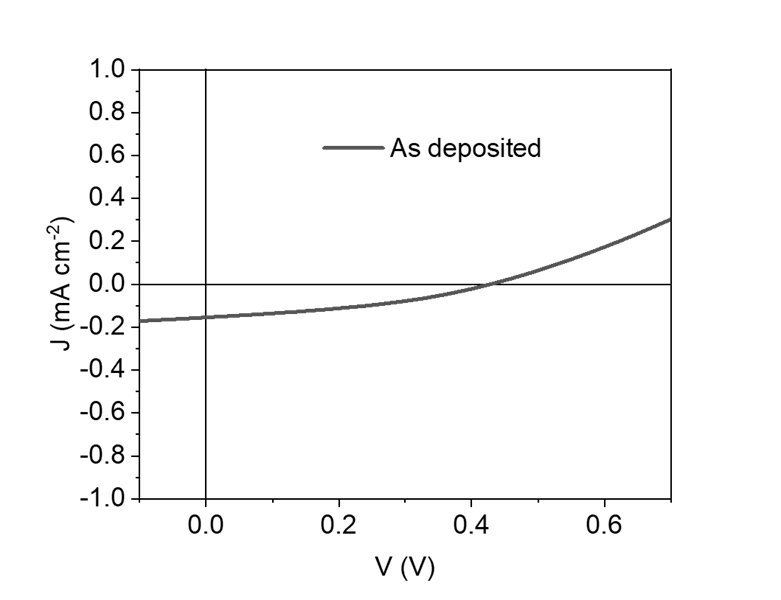


**Figure S15***. J-V* curve of solar cell based on Sb_2_S_3_ film without post-annealing.

**Table S10**. Device performance parameters of solar cell devices based on Sb_2_S_3_ films without post-annealing.

| Annealing | J_SC_ [mA cm^-2^] | V_OC_ [mV] | FF [%] | PCE [%] |
| --- | --- | --- | --- | --- |
| As deposited | 0.1 ± 0.01 (0.2) | 441 ± 9 (428) | 35.4 ± 0.5 (36.5) | 0.02 ± 0.002 (0.02) |
| Mean and SD of the top eight cells for each condition are reported. In parentheses, the parameters of the champion devices are reported. | | | | |


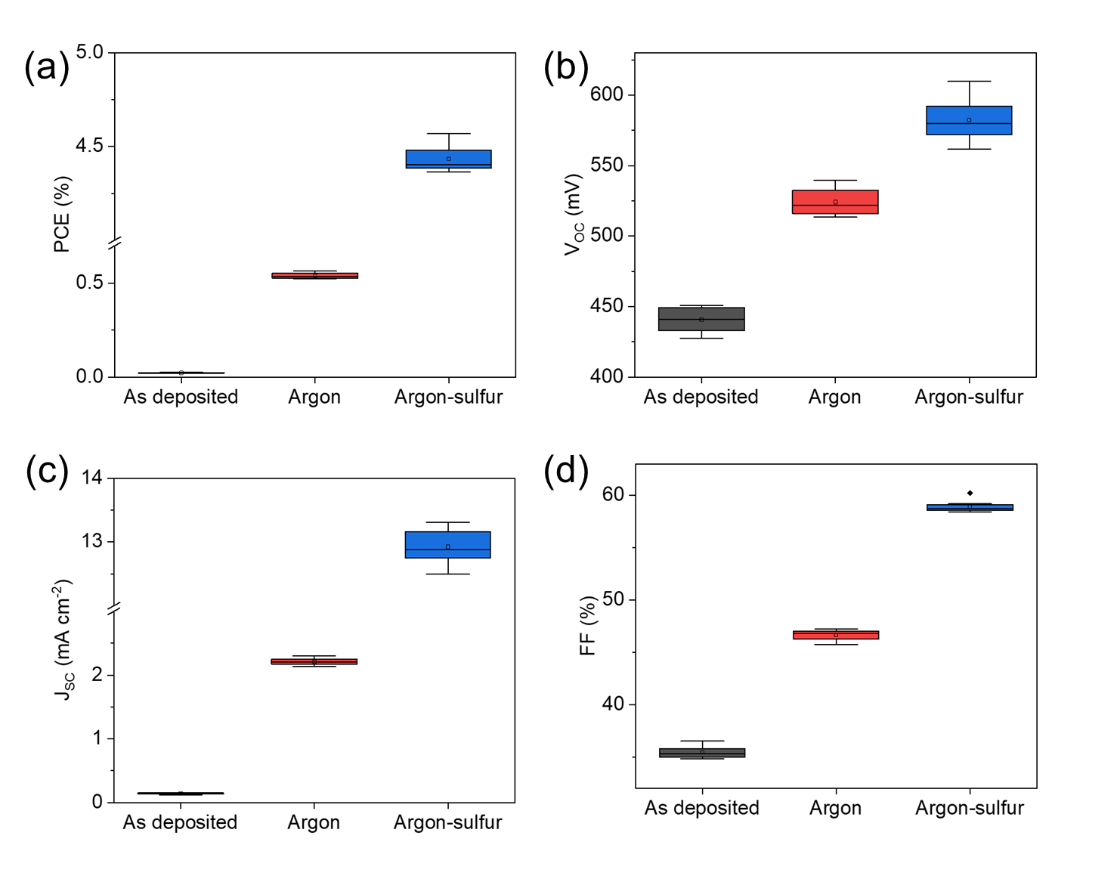


**Figure S16**. Statistical boxplots of photovoltaic parameters for Sb_2_S_3_ solar cells based on 120 nm Sb_2_S_3_ films; as-deposited, argon-annealed, and argon-sulfur annealed: (a) PCE, (b) V_OC_, (c) J_SC_, and (d) FF.

**Table S11**. Comparison of photovoltaic parameters for Sb_2_S_3_ solar cells based on 120 nm Sb_2_S_3_ films; as-deposited, argon-annealed, and argon-sulfur annealed: (a) PCE, (b) V_OC_, (c) J_SC_, and (d) FF.

| Annealing | J_SC_ [mA cm^-2^] | V_OC_ [mV] | FF [%] | PCE [%] |
| --- | --- | --- | --- | --- |
| As deposited | 0.16 (0.1 ± 0.0) | 427 (441 ± 9) | 36.50 (35.4 ± 0.5) | 0.02 (0.02 ±0.00) |
| Argon | 2.3 (2.2 ± 0.1) | 524 (524±9) | 46.8 (46.6 ± 0.5) | 0.6 (0.5 ± 0.0) |
| Argon-sulfur | 12.9 (12.9 ± 0.3) | 587 (582 ± 14) | 60.2 (58.9 ± 0.5) | 4.6 (4.4 ± 0.1) |
| ^a^Mean and standard deviation (SD), given in brackets, were calculated from the top eight devices. | | | | |


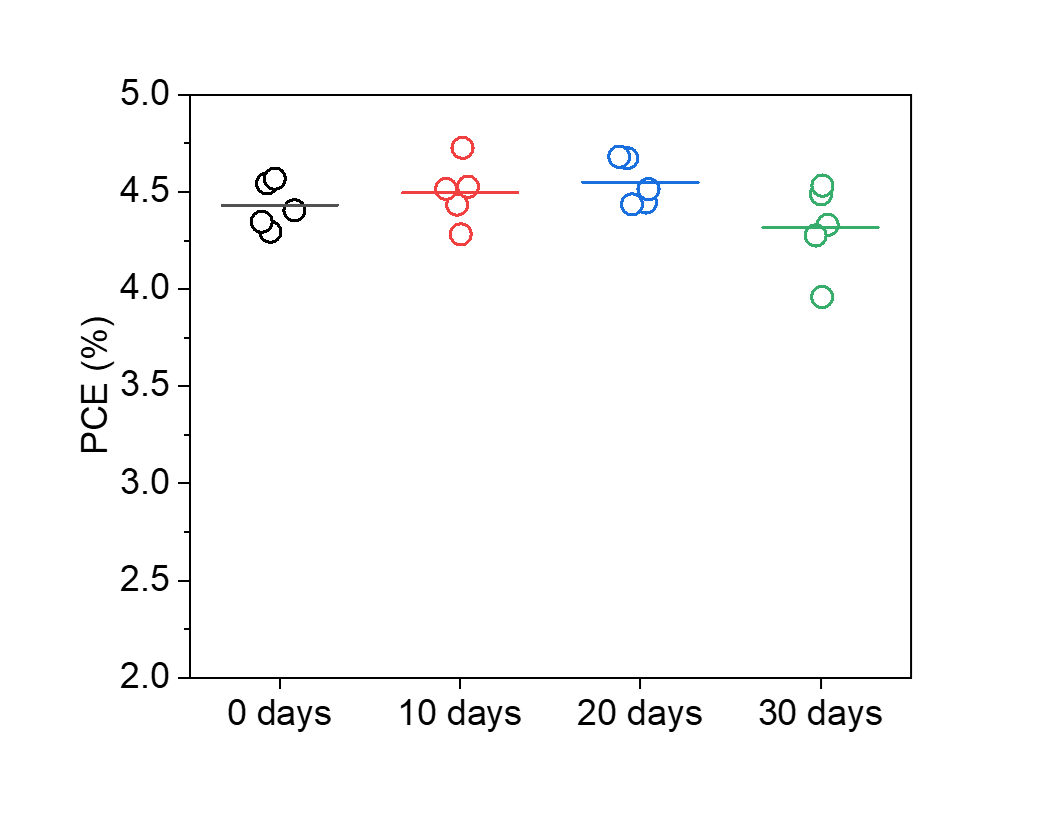


**Figure S17**. Stability of argon-sulfur-based champion devices in air. The devices were stored without encapsulation in the dark (relative humidity: 30-40% and temperature: 20-25 °C). Horizontal lines: mean PCE.


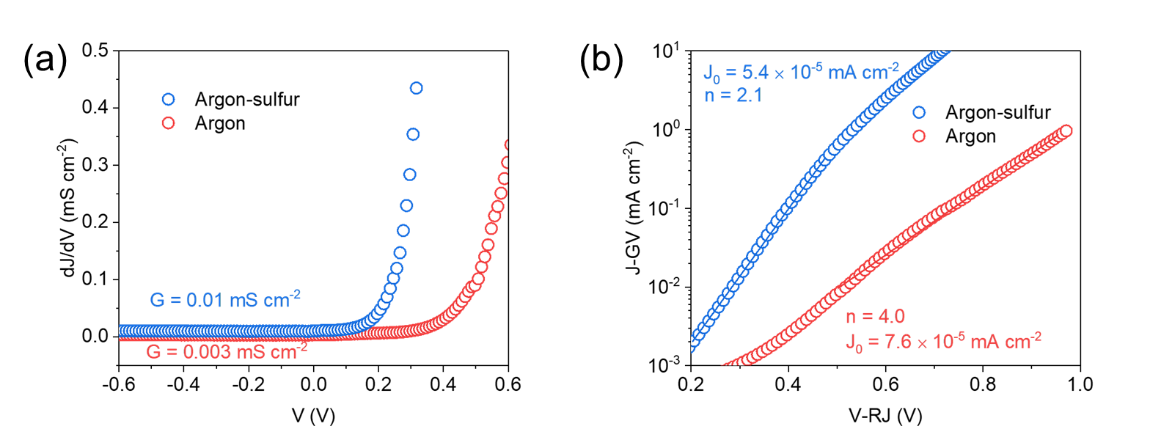


**Figure S18**. Dark currents. (a) d*J*/d*V* vs. *V* plot to calculate *G* (b) Semilogarithmic plot of (*J*+*J*_SC_–*GV*) against (*V*–*RJ*) with fit used to determine *J*_0_ and *n.*


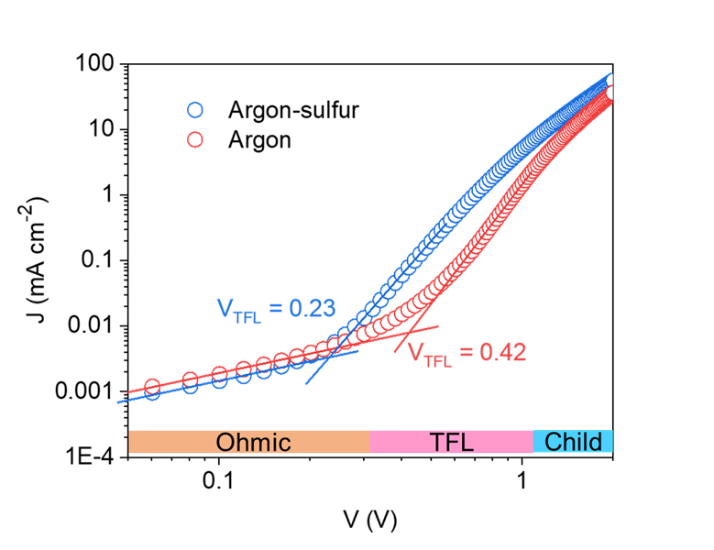


**Figure S19.** Logarithmic dark J-V curves of argon and argon-sulfur annealed devices, showing the ohmic, trap-fill limit, and Child regions.


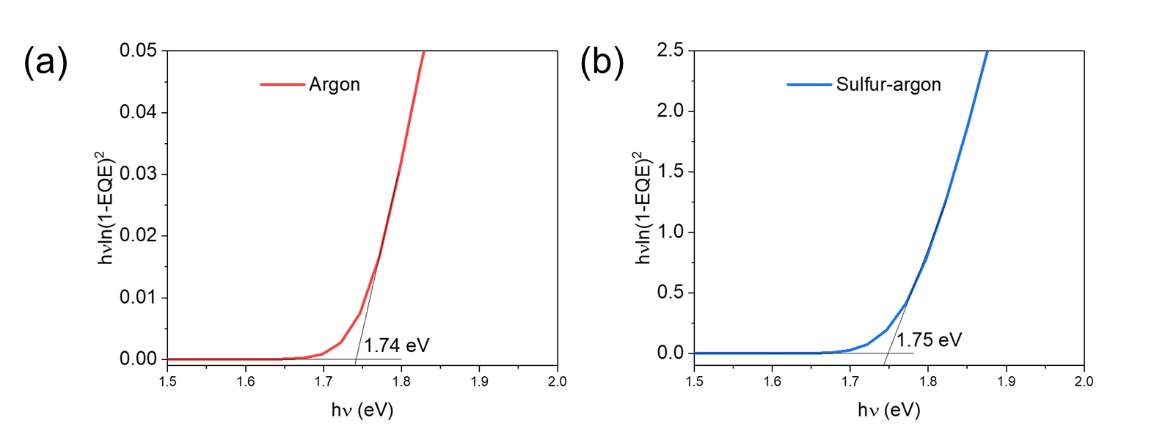


**Figure S20**. Bandgap estimation from EQE curves for Sb_2_S_3_ films annealed in (a) Argon and b) Argon-sulfur ambient.


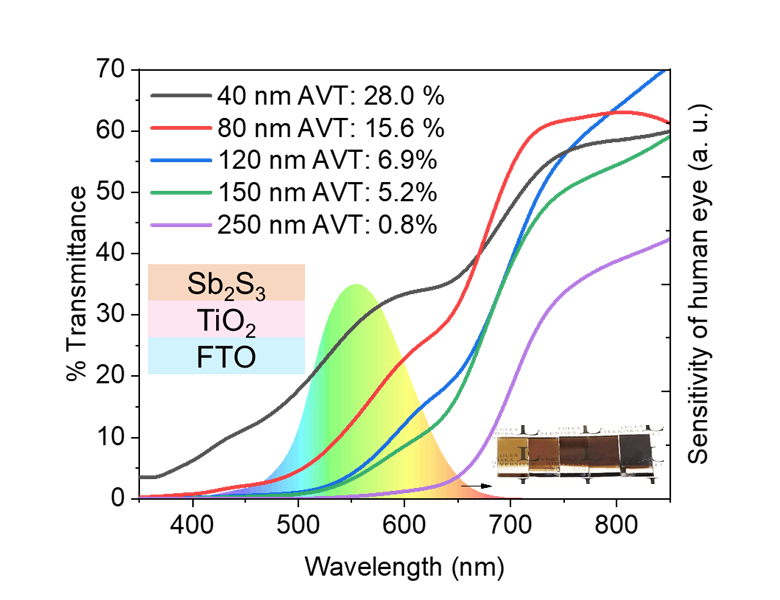


**Figure S21**. Transmittance vs. thickness of argon-sulfur annealed Sb_2_S_3_ films on FTO/TiO_2_. The sensitivity of the human eye and average visible transmittances (AVTs) are also given. Photographs of the films with increasing thickness from left to right are also shown.


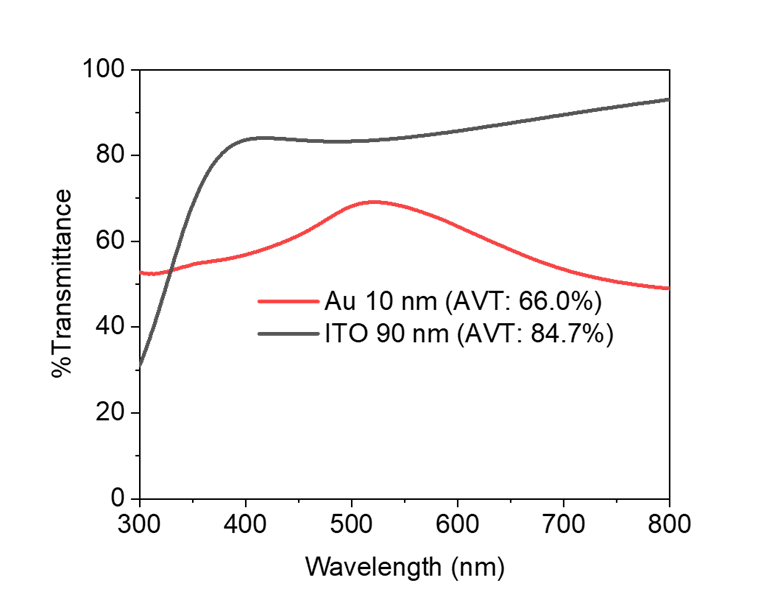


**Figure S22**. Transmittance of ~10 nm Au and 90 nm ITO sputtered on a glass substrate. AVTs are given in parentheses.

2.5


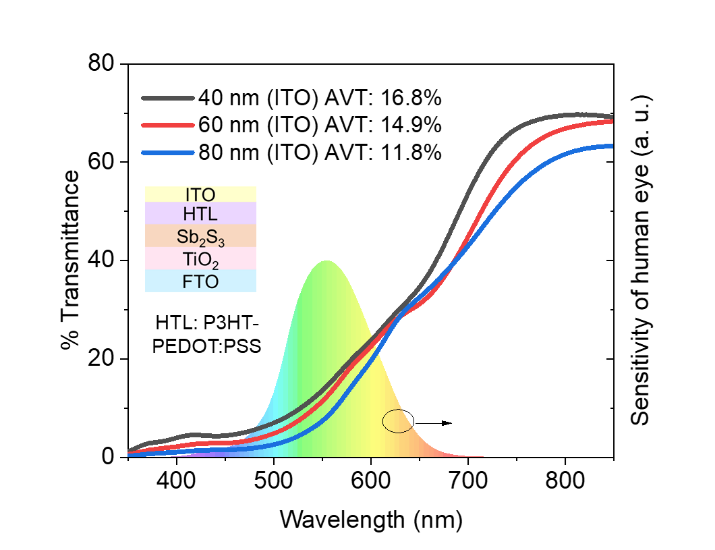


**Figure S23**. Transmittance curves for complete semitransparent devices using 75 nm ITO top electrode (solid lines). HTL: P3HT-PEDOT:PSS.


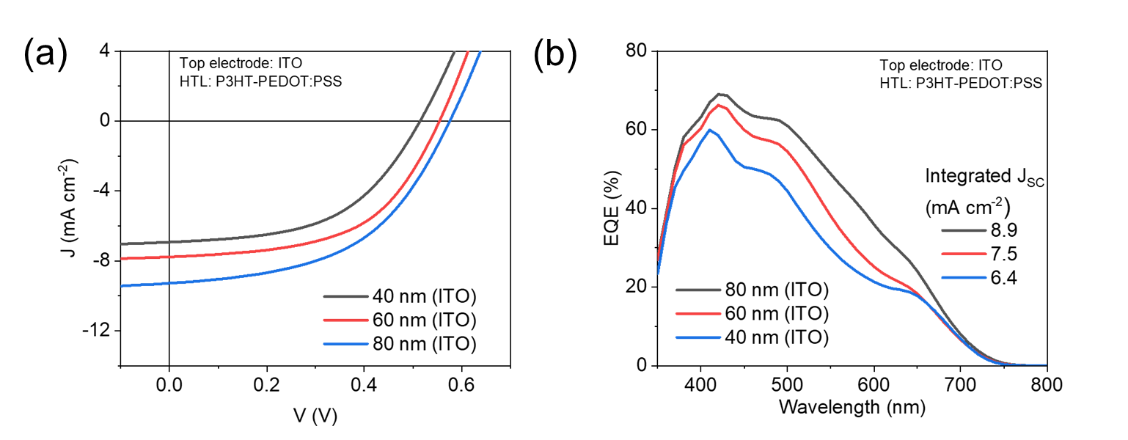


**Figure S24**. (a) J-V curves, and (b) EQE spectra of the devices with 40, 60, and 80 nm Sb_2_S_3_ absorber layers using ITO electrodes. HTL: P3HT-PEDOT:PSS


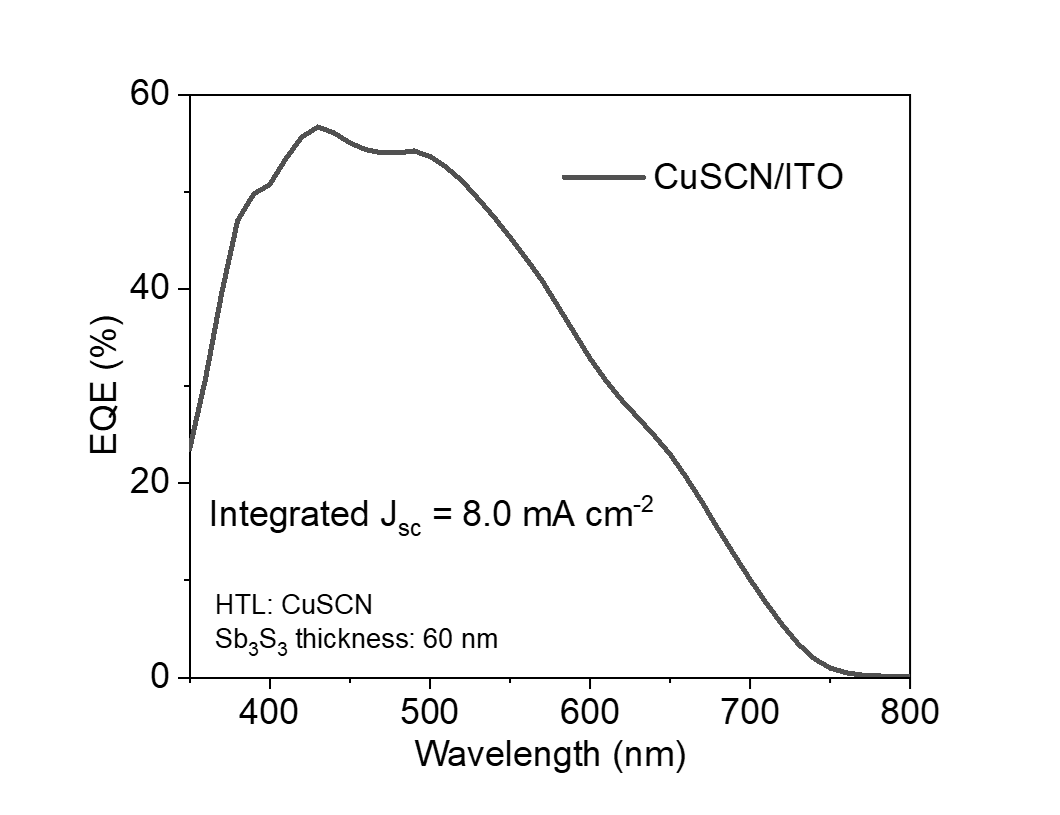


**Figure S25**. EQE spectra of CuSCN-based semitransparent solar cell using 60 nm Sb_2_S_3_ and ITO as the top transparent electrode.

**Table S12**. Device performance parameters of semitransparent Sb_2_S_3_ solar cell devices reported in the literature.

| Device structure | Method^a^ | d^b^  [nm] | J_SC_ [mA cm^-2^] | V_OC_ [mV] | FF [%] | PCE [%] | AVT^c^  [%] | LUE | Ref. |
| --- | --- | --- | --- | --- | --- | --- | --- | --- | --- |
| ITO/TiO_2_/Sb_2_S_3_/P3HT/PEDOT:PSS/Au(10 nm) | ALD | 85 | 12.1 | 679 | 42.0 | 3.4 | ~8 | ~0.27 | ^[6]^ |
| ITO/CdS/Sb_2_S_3_/Spiro-OMeTAD/PEDOT:PSS/graphene | HT | 100 | 10.0 | 509 | 42.7 | 2.2 | ~14 | ~0.31 | ^[7]^ |
| FTO/CdS/Sb_2_S_3_/CuSCN/Au (10 nm) | HT | 60 | 8.8 | 580 | 41.7 | 2.1 | 13.7 | 0.29 | ^[8]^ |
| FTO/CdS/Sb_2_S_3_/P3HT/PEDOT:PSS/Au(10 nm) | HT | 60 | 10.3 | 665 | 59.7 | 4.2 | 10.2 | 0.43 | ^[9]^ |
| FTO/TiO_2_-ZnS/Sb_2_S_3_/P3HT/PEDOT:PSS/Au(10 nm) | HT | 65 | 10.2 | 614 | 52.4 | 3.3 | 11.2 | 0.37 | ^[9]^ |
| FTO/TiO_2_/Sb_2_S_3_/P3HT-PEDOT:PSS/Au(10 nm) | Sputtering | 60 | 8.0 | 554 | 58.8 | 2.6 | 13.5 | 0.35 | This work |
| FTO/TiO_2_/Sb_2_S_3_/CuSCN/ITO | Sputtering | 60 | 8.4 | 516 | 47.0 | 2.0 | 20.5 | 0.41 | This work |
| ITO: Indium doped tin oxide; ALD: atomic layer deposition; ^a^Deposition method of Sb_2_S_3_ absorber layer; ^b^Thickness of the Sb_2_S_3_ absorber layer; ^c^estimated from the device transmittance curve if not mentioned. HT: hydrothermal. | | | | | | | | | |

**References:**

[1] Z. Wang, G.-J. Chen, R. Tang, Z.-H. Su, C.-Y. Duan, S.-M. Chen, Y.-L. Sun, S. Chen, G.-X. Liang, *Ceram. Int.* **2024**, *50*, 4729.

[2] S. Uc-Canché, E. Camacho-Espinosa, R. Mis-Fernández, M. Loeza-Poot, F. Ceh-Cih, J. L. Peña, *Materials* **2024**, *17*, 1656.

[3] J. Luo, W. Xiong, G. Liang, Y. Liu, H. Yang, Z. Zheng, X. Zhang, P. Fan, S. Chen, *J. Alloys Compd.* **2020**, *826*, 154235.

[4] H. Lei, T. Lin, X. Wang, P. Dai, Y. Guo, Y. Gao, D. Hou, J. Chen, Z. Tan, *J. Mater. Sci. Mater. Electron.* **2019**, *30*, 21106.

[5] C. Gao, J. Huang, H. Li, K. Sun, Y. Lai, M. Jia, L. Jiang, F. Liu, *Ceram. Int.* **2019**, *45*, 3044.

[6] S.-J. Lee, S.-J. Sung, K.-J. Yang, J.-K. Kang, J. Y. Kim, Y. S. Do, D.-H. Kim, *ACS Appl. Energy Mater.* **2020**, *3*, 12644.

[7] J. Zhang, W. Lian, Y. Yin, X. Wang, R. Tang, C. Qian, X. Hao, C. Zhu, T. Chen, *Sol. RRL* **2020**, *4*, 2000048.

[8] P. Kumar, M. Eriksson, D. S. Kharytonau, S. You, M. M. Natile, A. Vomiero, *ACS Appl. Energy Mater.* **2024**, *7*, 1421.

[9] P. Kumar, J. P. Thomas, D. S. Kharytonau, A. Gradone, N. Gilli, S. You, K. T. Leung, V. Morandi, A. Vomiero, *Nano Energy* **2025**, *134*, 110539.
